# Physiological effects of high-intensity versus low-intensity noninvasive positive pressure ventilation in patients with acute exacerbation of chronic obstructive pulmonary disease: a randomised controlled trial

**DOI:** 10.1186/s13613-022-01018-4

**Published:** 2022-05-19

**Authors:** Zujin Luo, Zhixin Cao, Yichong Li, Jiawei Jin, Wei Sun, Jian Zhu, Na Zhao, Jichen Liu, Bing Wei, Yue Hu, Ying Zhang, Yingmin Ma, Chen Wang

**Affiliations:** 1 Xuanwu Hospital, Capital Medical University, No. 45 Changchun Street, Xicheng District, Beijing, 100053 China; 2 Department of Respiratory and Critical Care Medicine, Beijing Institute of Respiratory Medicine, Beijing Chao-Yang Hospital, Capital Medical University, No. 5 Jingyuan Road, Shijingshan District, Beijing, 100043 China; 3 Department of Clinical Research and Epidemiology, Fuwai Hospital Chinese Academy of Medical Sciences, No. 12 Lanshan Road, Nanshan District, Shenzhen, 518057 Guangdong Province China; 4 Department of Emergency Medicine, Beijing Chao-Yang Hospital Western Branch, Capital Medical University, No. 5 Jingyuan Road, Shijingshan District, Beijing, 100043 China; 5 Department of Respiratory and Critical Care Medicine, Beijing Youan Hospital, Capital Medical University, No. 8, Xi Tou Tiao, Youanmen wai, Fengtai District, Beijing, 100069 China; 6 Department of Pulmonary and Critical Care Medicine, China-Japan Friendship Hospital, No. 2 Yinghua East Street, Chaoyang District, Beijing, 100029 China; 7 National Clinical Research Center for Respiratory Diseases, Beijing, China; 8 Chinese Academy of Medical Sciences and Peking Union Medical College, Beijing, China; 9 Department of Respiratory Medicine, Capital Medical University, Beijing, China

**Keywords:** Noninvasive positive pressure ventilation, High intensity, Low intensity, Chronic obstructive pulmonary disease, Exacerbation, Hypercapnia, Normocapnia, Physiological effects

## Abstract

**Background:**

High-intensity noninvasive positive pressure ventilation (NPPV) is a novel ventilatory approach to maximally decreasing elevated arterial carbon dioxide tension (PaCO_2_) toward normocapnia with stepwise up-titration of pressure support. We tested whether high-intensity NPPV is more effective than low-intensity NPPV at decreasing PaCO_2_, reducing inspiratory effort, alleviating dyspnoea, improving consciousness, and improving NPPV tolerance in patients with acute exacerbation of chronic obstructive pulmonary disease (AECOPD).

**Methods:**

In this physiological, randomised controlled trial, we assigned 24 AECOPD patients to undergo either high-intensity NPPV (*n* = 12) or low-intensity NPPV (*n* = 12). The primary outcome was PaCO_2_ 24 h after randomisation. Secondary outcomes included gas exchange other than PaCO_2_ 24 h after randomisation, inspiratory effort, dyspnoea, consciousness, NPPV tolerance, patient–ventilator asynchrony, cardiac function, ventilator-induced lung injury (VILI), and NPPV-related adverse events.

**Results:**

Inspiratory positive airway pressure 24 h after randomisation was significantly higher (28.0 [26.0–28.0] vs. 15.5 [15.0–17.5] cmH_2_O; p = 0.000) and NPPV duration within the first 24 h was significantly longer (21.8 ± 2.1 vs. 15.3 ± 4.7 h; p = 0.001) in the high-intensity NPPV group. PaCO_2_ 24 h after randomisation decreased to 54.0 ± 11.6 mmHg in the high-intensity NPPV group but only decreased to 67.4 ± 10.6 mmHg in the low-intensity NPPV group (p = 0.008). Inspiratory oesophageal pressure swing, oesophageal pressure–time product (PTPes)/breath, PTPes/min, and PTPes/L were significantly lower in the high-intensity group. Accessory muscle use and dyspnoea score 24 h after randomisation were also significantly lower in that group. No significant between-groups differences were observed in consciousness, NPPV tolerance, patient–ventilator asynchrony, cardiac function, VILI, or NPPV-related adverse events.

**Conclusions:**

High-intensity NPPV is more effective than low-intensity NPPV at decreasing elevated PaCO_2_, reducing inspiratory effort, and alleviating dyspnoea in AECOPD patients.

*Trial registration*: ClinicalTrials.gov (NCT04044625; registered 5 August 2019).

**Supplementary Information:**

The online version contains supplementary material available at 10.1186/s13613-022-01018-4.

## Background

Noninvasive positive pressure ventilation (NPPV) has been increasingly used in the care of patients with acute exacerbation of chronic obstructive pulmonary disease (AECOPD) based on several lines of supporting evidence [1–7]. However, low-intensity NPPV, which uses a relatively low inspiratory positive airway pressure (IPAP; typically < 18 cmH_2_O), is normally used [6–9]. The fact that NPPV fails in approximately 15% of patients with AECOPD may be partly associated with inadequate pressure support and limited improvement in alveolar ventilation due to low-intensity NPPV [1, 6–12].

High-intensity NPPV, a form of pressure-limited ventilation in which IPAP levels typically range from 20 to 30 cmH_2_O, was introduced as a novel ventilatory approach to maximally decrease elevated arterial carbon dioxide tension (PaCO_2_) toward normocapnia with stepwise up-titration of IPAP [9, 13, 14]. In theory, high-intensity NPPV may be more effective than low-intensity NPPV at augmenting alveolar ventilation and offsetting the extra dead space caused by the face mask and may better reduce inspiratory effort and alleviate dyspnoea, resulting in greater NPPV tolerance. Several positive results have been reported for the use of high-intensity NPPV to treat stable hypercapnic chronic obstructive pulmonary disease (COPD) [15–17]. For example, it has been found that high-intensity NPPV is superior to low-intensity NPPV at reducing inspiratory effort and improving gas exchange, lung function, patient tolerance, and health-related quality of life in such patients [15, 16]. However, to date no data are available on whether high-intensity NPPV is superior to low-intensity NPPV in patients with AECOPD.

In this physiological trial, we tested the hypothesis that high-intensity NPPV would be more effective than low-intensity NPPV at decreasing elevated PaCO_2_, reducing inspiratory effort, alleviating dyspnoea, improving consciousness, and improving NPPV tolerance in patients with AECOPD.

## Methods

### Trial design

This prospective, randomised controlled trial (RCT) was conducted in the 12-bed respiratory intensive care unit (ICU) of Beijing Chao-Yang Hospital Western Branch in China. The protocol was approved by the ethics committee at Beijing Chao-Yang Hospital (reference no. 2019-KE-263), and written informed consent was obtained from all patients, their next of kin, or other surrogate decision-makers as appropriate. The trial was registered with clinicaltrials.gov (identifier: NCT04044625).

### Patients

We screened all COPD patients admitted to the respiratory ICU. Patients were considered eligible for the trial if they had been diagnosed with AECOPD as defined by the 2019 criteria of the Global Initiative for Chronic Obstructive Lung Disease [18], had arterial pH < 7.35 and PaCO_2_ > 45 mmHg at ICU admission, and still had PaCO_2_ > 45 mmHg after a 6 h screening period while receiving low-intensity NPPV. Exclusion criteria are provided in Additional file 1: Supplementary methods.

### Randomisation and blinding

Randomisation was accomplished by a computer-generated random number sequence. Each allocation sequence was concealed through the use of numbered, opaque, sealed envelopes until the intervention assignment was finished and was managed by an independent employee who was not involved in the trial. Eligible patients were assigned at a 1:1 ratio to undergo either high-intensity NPPV or low-intensity NPPV. At least two investigators per patient conducted the study: One performed the intervention defined in the protocol, and the other performed the outcome measurements. All data analyses were performed by the trial statistician, who was not involved in the trial.

### Interventions

In the high-intensity NPPV group, IPAP was initially adjusted in increments/decrements of 1–2 cmH_2_O, typically ranging from 20 to 30 cmH_2_O (or a tolerated maximum), to obtain a tidal volume (*V*_T_) 10–15 mL/kg of predicted body weight (PBW) and a respiratory rate (RR) < 25 breaths/min. Subsequent adjustments to IPAP were based on the results of arterial blood gases (ABGs; up to 30 cmH_2_O) to achieve either normocapnia (if possible) or a maximal reduction in PaCO_2_. If PaCO_2_ decreased to less than 35 mmHg, IPAP was decreased to achieve normocapnia. Patients were encouraged to use NPPV as continuously as possible, but brief disconnection from the ventilator was allowed to clear secretions, drink water, or eat.

In the low-intensity NPPV group, as well as during the 6-h screening period, IPAP was initially adjusted in increments/decrements of 1–2 cmH_2_O (up to 20 cmH_2_O), according to patients’ tolerance, to obtain a V_T_ 6–10 mL/kg of PBW and an RR < 25 breaths/min. Subsequent adjustments to IPAP were based on the results of ABGs (up to 20 cmH_2_O) to achieve a pH of ≥ 7.35 and to reduce PaCO_2_ to an extent accepted by the attending physician. Patients were encouraged to use NPPV as much as possible during the first 6 h after randomisation and at least 10 h per day. Brief disconnection from the ventilator was allowed to clear secretions, drink water, or eat but was not scheduled.

In both groups, expiratory positive airway pressure (EPAP) was set at 5–8 cmH_2_O, the backup RR was set at 12 breaths/min, the inspiratory time was set at 0.8–1.2 s, the rise slope was set at level 1 or 2, and fraction of inspired oxygen (FiO_2_) was adjusted to obtain an oxygen saturation measured by pulse oximetry of 90–95%. All patients used the same noninvasive ventilator (Respironics V60, Philips Respironics, Carlsbad, CA, USA) in the bilevel positive airway pressure (spontaneous/timed) mode. An oronasal mask (Philips Respironics) was used as a first choice, but a nasal mask (Philips Respironics) was optional if patients did not tolerate the oronasal mask.

Medical treatments other than NPPV were based on the 2019 guidelines of the Global Initiative for Chronic Obstructive Lung Disease [18] and routine clinical practice in the respiratory ICU. If severe alkalosis occurred (pH > 7.55), arginine was provided.

### Outcome measurements

The primary outcome was PaCO_2_ 24 h after randomisation. Secondary outcomes included gas exchange other than PaCO_2_ 24 h after randomisation, inspiratory effort, dyspnoea, consciousness, NPPV tolerance, patient–ventilator asynchrony, cardiac function, ventilator-induced lung injury (VILI), and NPPV-related adverse events.

For gas exchange, we recorded pH, arterial oxygen tension [PaO_2_], PaCO_2_, and bicarbonates at baseline and 2, 6, 24, 48, and 72 h after randomisation, and calculated the differences in PaCO_2_ between baseline and these other time points, respectively.

For inspiratory effort, we measured inspiratory oesophageal pressure swing (ΔPes), oesophageal pressure–time product (PTPes)/breath, PTPes/min, and PTPes/L over the last 3–5 min of oesophageal pressure (Pes) recording within 24 h after randomisation (see Additional file 1: Supplementary methods for more details).

For dyspnoea, consciousness, and NPPV tolerance, we recorded accessory muscle use, dyspnoea score, Glasgow Coma Scale (GCS) score, Kelly–Matthay score, and NPPV tolerance score at baseline and 2, 6, 24, 48, and 72 h after randomisation (see Additional file 1: Supplementary methods for more details).

All asynchrony events (including ineffective efforts, auto-triggering, double-triggering, premature cycling, and delayed cycling) were determined by visual inspection of the tracings of Pes, airway pressure, and flow over the last 10 min of these recordings within 24 h after randomisation, and an asynchrony index was computed (see Additional file 1: Supplementary methods and Fig. S1 for more details).

Regarding cardiac function, we recorded heart rate and blood pressure; measured serum N-terminal pro-B-type natriuretic peptide, troponin I, and creatine kinase isoenzyme; and performed bedside echocardiographic examination at baseline and 24, 28, and 72 h after randomisation.

To assess VILI, we measured plasma levels of VILI-related inflammatory mediators, including tumour necrosis factor (TNF)-α, interleukin (IL)-1β, IL-6, IL-8, IL-10, and macrophage inflammatory protein (MIP)-2 at baseline and 24, 28, and 72 h after randomisation (see Additional file 1: Supplementary methods for more details).

### Statistical analysis

We expected that the mean (± standard deviation [SD]) PaCO_2_ 24 h after randomisation would be 65 ± 15 mmHg in the low-intensity NPPV group, based on our clinical experience and previous studies [1, 6–8, 19]. Based on the assumption that mean PaCO_2_ 24 h after randomisation would be 45 ± 15 mmHg in the high-intensity NPPV group, a sample of 12 patients in each group was required to detect an absolute between-groups difference of 20 mmHg in PaCO_2_ 24 h after randomisation. We used a superiority test to compare the means of the two groups, with a superiority margin of 3 mmHg, 85% power, and a one-tailed alpha of 0.05.

Continuous variables are presented as means ± SD with normal distributions or as medians (25th–75th percentiles) with non-normal distributions unless otherwise specified. Qualitative or categorical variables are presented as absolute frequencies with percentages. The test of normality was performed with the Kolmogorov–Smirnov test, and the test of homogeneity of variances was performed with Levene’s test. Continuous variables were compared between the two groups with Student’s t test for normally distributed variables and the Mann–Whitney U test for non-normally distributed variables. Qualitative or categorical variables were compared with the Fisher’s exact test. All tests were two sided. Differences with *p* < 0.05 were considered statistically significant. Statistical analyses were performed with SPSS (version 25.0; IBM, Chicago, IL, USA).

## Results

### Patients

**Fig. 1:**
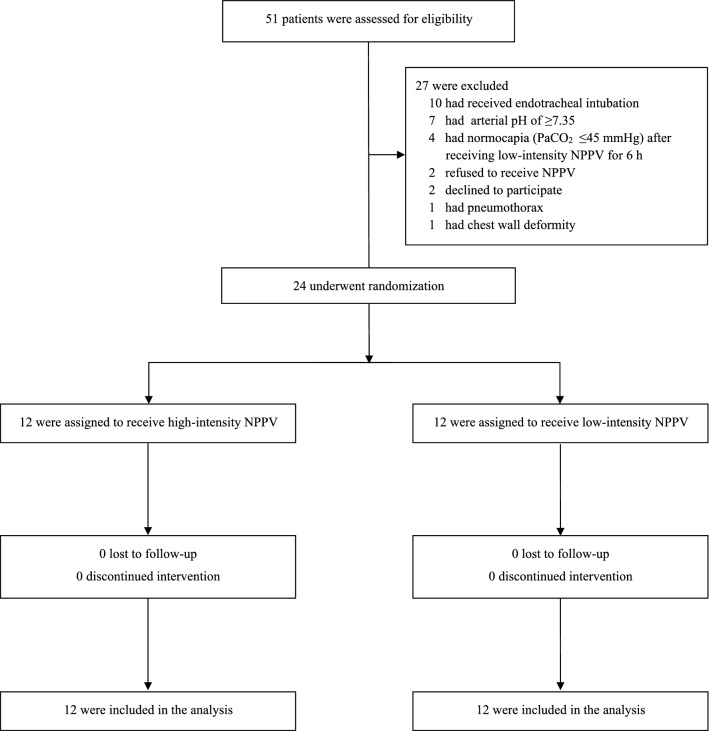
Screening, randomisation, and analysis. *PaCO*_*2*_ arterial carbon dioxide tension, *NPPV* noninvasive positive pressure ventilation

From September 2019 through June 2021, a total of 51 patients with AECOPD were eligible for inclusion in the trial. Of these patients, 27 were excluded and 24 ultimately underwent randomisation. A total of 12 patients were assigned to the high-intensity NPPV group, 12 were assigned to the low-intensity NPPV group, and all 24 were included in the analysis (Fig. 1). Both groups had similar characteristics at baseline (Table 1 and Additional file 1: Table S1). There were no significant between-groups differences in lung function variables or PaCO_2_ at ICU admission and at randomisation.

**Table 1:** Baseline characteristics of the patients

Characteristic	High-intensity NPPV (*n* = 12)	Low-intensity NPPV (*n* = 12)	*p* value
Demographics			
Age, years	72.3 ± 9.0	70.8 ± 8.2	0.608
Male, n (%)	8 (66.7)	8 (66.7)	> 0.999
Height, cm	169.0 (165.0–170.0)	165.0 (160.0–172.8)	0.906
Predicted body weight, kg	63.7 (57.2–66.0)	61.5 (53.1–68.5)	0.838
Actual body weight, kg	64.6 ± 13.0	67.8 ± 16.3	0.605
Body mass index, kg/cm^2^	23.0 ± 4.3	24.3 ± 5.4	0.538
Smoking history			
Former smoker, n (%)	5 (41.7)	9 (75.0)	0.214
Current smoker, n (%)	3 (25.0)	3 (25.0)	> 0.999
Smoking index, pack·year	30.0 (12.5–54.4)	25.0 (10.0–38.1)	0.663
Lung function			
FEV_1_, L	0.5 ± 0.1	0.6 ± 0.2	0.087
FEV_1_, % of predicted value	21.8 ± 6.5	27.1 ± 10.2	0.145
FVC, L	1.4 ± 0.3	1.7 ± 0.6	0.129
FVC, % of predicted value	43.9 ± 8.3	53.7 ± 15.2	0.066
FEV_1_/FVC, %	38.0 ± 14.4	38.9 ± 14.9	0.888
FEV_1_/FVC, % of predicted value	54.0 ± 18.9	51.8 ± 22.1	0.792
Within the previous 1 year, n (%)	2 (16.7)	5 (41.7)	0.371
At ICU discharge, n (%)	10 (83.3)	7 (58.3)	0.371
Reason for acute exacerbation			
Respiratory infection, n (%)	7 (58.3)	7 (58.3)	> 0.999
Acute heart failure, n (%)	2 (16.7)	1 (8.3)	> 0.999
Exposure to air pollutants, n (%)	1 (8.3)	1 (8.3)	> 0.999
Undetermined, n (%)	2 (16.7)	3 (25.0)	> 0.999
Arterial blood gas at ICU admission			
pH	7.26 ± 0.07	7.29 ± 0.04	0.220
PaCO_2_, mmHg	90.7 ± 12.8	87.2 ± 17.9	0.584
PaO_2_/FiO_2_, mmHg	214 ± 46	189 ± 54	0.219
Bicarbonates, mmol/L	35.6 ± 5.9	36.0 ± 5.4	0.881
Arterial blood gas at randomisation			
pH	7.31 ± 0.07	7.33 ± 0.06	0.441
PaCO_2_, mmHg	83.4 ± 12.3	78.6 ± 11.3	0.331
PaO_2_/FiO_2_, mmHg	231 ± 63	240 ± 60	0.701
Bicarbonates, mmol/L	37.2 ± 7.6	37.2 ± 5.7	0.993

Data are presented as means ± standard deviations, medians (25th–75th percentiles), or frequencies (percentages) of patients as appropriate

*NPPV* noninvasive positive pressure ventilation, *FEV*_*1*_ forced expiratory volume in 1 s, *FVC* forced vital capacity, ICU intensive care unit, *PaCO*_*2*_ arterial carbon dioxide tension, *PaO*_*2*_ arterial oxygen tension, *FiO*_*2*_ fraction of inspired oxygen

### NPPV setting, monitoring, and recording

**Fig. 2:**
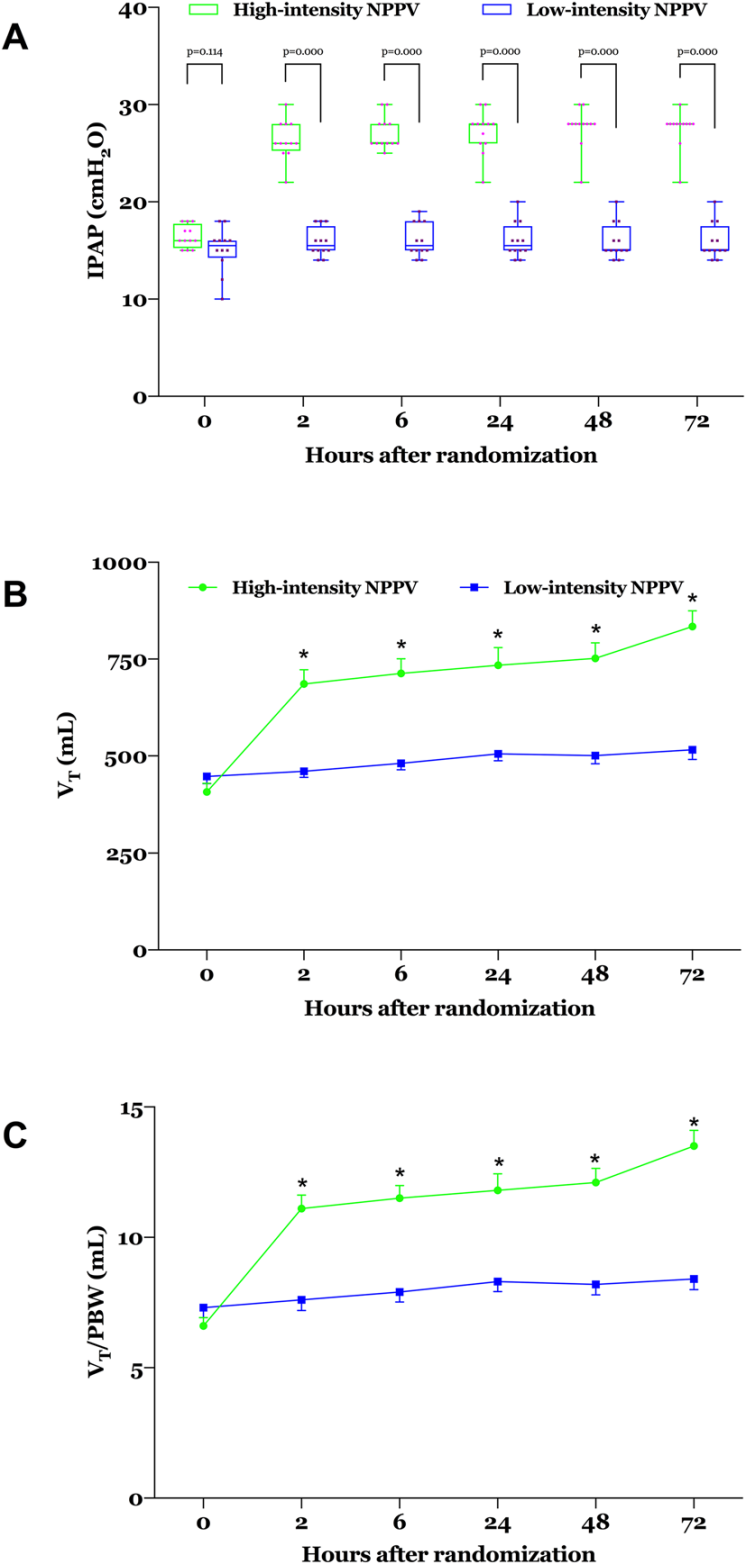
**a** IPAP, **b** V_T_, and **c** V_T_/PBW from baseline through 72 h after randomisation. Values are individuals, medians, and interquartile ranges with whiskers indicating minimum and maximum for IPAP and means and standard errors for V_T_ and V_T_/PBW. *p < 0.05 between the two groups at the same time. *NPPV* noninvasive positive pressure ventilation, *IPAP* inspiratory positive airway pressure, *V*_T_ tidal volume, *PBW* predicted body weight

As shown in Fig. 2 and Additional file 1: Table S2, the ventilator settings and monitored parameters at randomisation did not differ significantly between the two groups. IPAP, V_T_, and minute volume were significantly higher, and RR was significantly lower, 24 h after randomisation in the high-intensity NPPV group than in the low-intensity NPPV group. V_T_ 24 h after randomisation was above 10 mL/kg of PBW in the high-intensity NPPV group but was below that in the low-intensity NPPV group. EPAP, FiO_2_, and leakage 24 h after randomisation did not differ significantly between the two groups. Duration of NPPV within the first 24 h was significantly longer in the high-intensity NPPV group than in the low-intensity NPPV group (21.8 ± 2.1 vs. 15.3 ± 4.7 h; p = 0.001). More details on NPPV setting, monitoring, and recording over the first 72 h are provided in Fig. 2 and Additional file 1: Table S2.

### Gas exchange, inspiratory effort, dyspnoea, consciousness, and NPPV tolerance

**Fig. 3:**
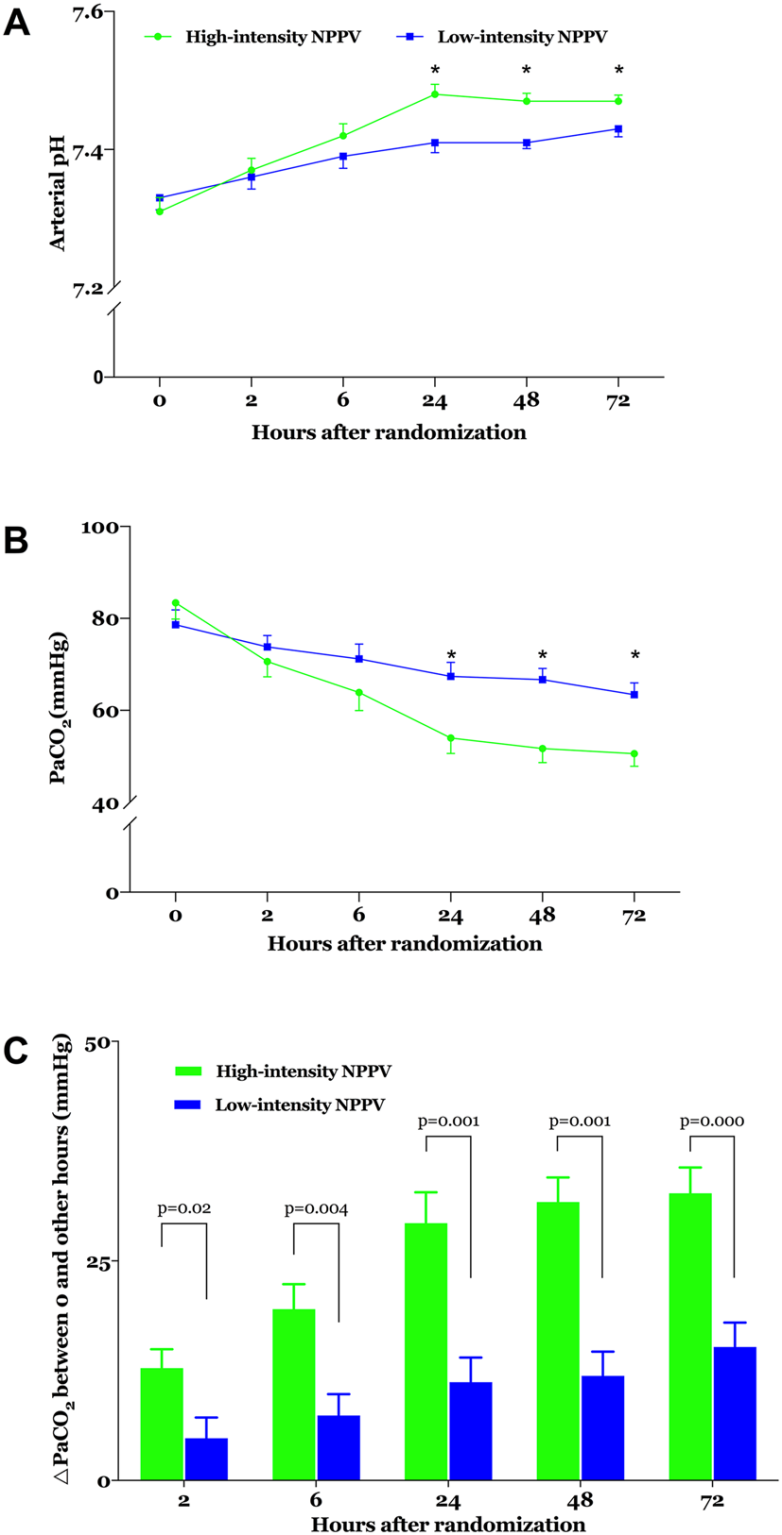
**a** pH and **b** PaCO_2_ from baseline through 72 h after randomisation and **c** differences in PaCO_2_ between baseline and other times after randomisation. Values are means and standard errors. *p < 0.05 between the two groups at the same time. *NPPV* noninvasive positive pressure ventilation, *PaCO*_*2*_ arterial carbon dioxide tension.

PaCO_2_ was significantly lower and pH was significantly higher 24 h after randomisation in the high-intensity NPPV group than in the low-intensity NPPV group (Fig. 3; Table 2). PaCO_2_ 24 h after randomisation, the primary outcome, decreased to 54.0 ± 11.6 mmHg in the high-intensity NPPV group but only decreased to 67.4 ± 10.6 mmHg in the low-intensity NPPV group (*p* = 0.008). During the first 24 h after randomisation, four patients in the high-intensity NPPV group achieved normocapnia whereas none in the low-intensity NPPV group did so (*p* = 0.093). Compared to the low-intensity NPPV group, the high-intensity NPPV group had greater differences in PaCO_2_ between baseline and 24 h after randomisation (Fig. 3). PaO_2_/FiO_2_ and bicarbonates 24 h after randomisation did not differ significantly between the two groups (Table 2). More details on gas exchange over the first 72 h are provided in Fig. 3 and Table 2.

**Table 2:** Gas exchange, inspiratory effort, dyspnoea, consciousness, and NPPV tolerance

Variable	High-intensity NPPV (*n* = 12)	Low-intensity NPPV (*n* = 12)	*p* value
Gas exchange			
pH			
Baseline	7.31 ± 0.07	7.33 ± 0.06	0.441
2 h	7.37 ± 0.06	7.36 ± 0.06	0.603
6 h	7.42 ± 0.06	7.39 ± 0.06	0.202
24 h	7.48 ± 0.05	7.41 ± 0.05	0.002
48 h	7.47 ± 0.04	7.41 ± 0.03	0.001
72 h	7.47 ± 0.03	7.43 ± 0.04	0.013
PaCO_2_, mmHg			
Baseline	83.4 ± 12.3	78.6 ± 11.3	0.331
2 h	70.6 ± 11.4	73.8 ± 8.6	0.445
6 h	63.9 ± 13.6	71.2 ± 11.2	0.166
24 h	54.0 ± 11.6	67.4 ± 10.6	0.008
48 h	51.7 ± 10.6	66.7 ± 8.4	0.001
72 h	50.6 ± 9.5	63.4 ± 8.9	0.003
Difference in PaCO_2_, mmHg			
Between baseline and 2 h	12.8 ± 7.4	4.8 ± 8.2	0.020
Between baseline and 6 h	19.5 ± 9.9	7.4 ± 8.4	0.004
Between baseline and 24 h	29.3 ± 12.2	11.2 ± 9.7	0.001
Between baseline and 48 h	31.7 ± 9.7	11.9 ± 9.6	0.000
Between baseline and 72 h	32.7 ± 10.2	15.2 ± 9.6	0.000
PaO_2_/FiO_2_, mmHg			
Baseline	230 ± 63	240 ± 60	0.701
2 h	247 ± 60	232 ± 60	0.549
6 h	273 ± 71	253 ± 79	0.523
24 h	300 ± 92	256 ± 60	0.187
48 h	298 ± 66	257 ± 81	0.186
72 h	306 ± 72	269 ± 58	0.179
Bicarbonates, mmol/L			
Baseline	37.2 ± 7.6	37.2 ± 5.7	0.993
2 h	37.4 ± 7.5	38.4 ± 6.4	0.715
6 h	38.7 ± 7.7	39.1 ± 5.4	0.894
24 h	38.4 ± 6.2	39.4 ± 5.3	0.670
48 h	36.6 ± 5.3	38.6 ± 5.7	0.384
72 h	35.7 ± 5.7	38.8 ± 4.7	0.156
Inspiratory effort*			
ΔPes, cmH_2_O	2.8 ± 2.2 (n = 11)	6.2 ± 4.5 (n = 11)	0.031
PTPes/breath, cmH_2_O·s	1.3 ± 1.3 (n = 11)	4.4 ± 2.2 (n = 11)	0.001
PTPes/min, cmH_2_O·s/min	20.1 ± 17.9 (n = 11)	83.3 ± 54.9 (n = 11)	0.003
PTPes/L, cmH_2_O·s/L	1.7 ± 1.4 (n = 11)	9.2 ± 5.8 (n = 11)	0.001
Dyspnoea, consciousness, and NPPV tolerance			
Accessory muscle use			
Baseline	4.0 (4.0–5.0)	4.0 (3.0–4.8)	0.387
2 h	3.0 (2.0–3.0)	3.5 (3.0–4.0)	0.097
6 h	2.0 (2.0–3.0)	3.0 (3.0–3.0)	0.008
24 h	2.0 (1.0–2.0)	3.0 (2.0–3.0)	0.003
48 h	1.0 (1.0–2.0)	2.0 (2.0–3.0)	0.004
72 h	1.0 (1.0–2.0)	3.0 (2.0–2.8)	0.024
Dyspnoea score			
Baseline	8.0 (7.3–10.0)	8.0 (6.0–8.0)	0.060
2 h	5.0 (3.3–6.0)	6.0 (4.0–7.8)	0.306
6 h	3.0 (2.0–5.5)	4.5 (3.3–6.0)	0.157
24 h	1.0 (1.0–2.8)	3.5 (2.3–5.0)	0.004
48 h	1.0 (0.0–2.0)	2.0 (2.0–3.8)	0.009
72 h	1.0 (0.0–1.8)	2.0 (2.0–2.0)	0.001
GCS score			
Baseline	14.0 (12.0–15.0)	15.0 (14.3–15.0)	0.092
2 h	15.0 (15.0–15.0)	15.0 (15.0–15.0)	0.929
6 h	15.0 (15.0–15.0)	15.0 (15.0–15.0)	> 0.999
24 h	15.0 (15.0–15.0)	15.0 (15.0–15.0)	0.317
48 h	15.0 (15.0–15.0)	15.0 (15.0–15.0)	> 0.999
72 h	15.0 (15.0–15.0)	15.0 (15.0–15.0)	> 0.999
Kelly–Matthay score			
Baseline	3.0 (2.0–3.0)	2.0 (2.0–2.8)	0.178
2 h	1.5 (1.0–2.0)	2.0 (1.0–2.0)	0.754
6 h	1.0 (1.0–2.0)	1.5 (1.0–2.0)	0.514
24 h	1.0 (1.0–1.0)	1.0 (1.0–2.0)	0.320
48 h	1.0 (1.0–1.0)	1.0 (1.0–1.0)	0.546
72 h	1.0 (1.0–1.0)	1.0 (1.0–1.0)	> 0.999
NPPV tolerance score			
Baseline	3.0 (2.3–4.0)	4.0 (3.0–4.0)	0.415
2 h	3.5 (3.0–4.0)	4.0 (3.0–4.0)	> 0.999
6 h	4.0 (3.0–4.0)	4.0 (3.0–4.0)	0.741
24 h	4.0 (3.0–4.0)	4.0 (3.0–4.0)	0.500
48 h	4.0 (3.0–4.0)	4.0 (3.0–4.0)	0.741
72 h	4.0 (3.3–4.0)	4.0 (3.0–4.0)	0.297

Data are presented as means ± standard deviations, medians (25th–75th percentiles), or frequencies (percentages) of patients as appropriate

*NPPV* noninvasive positive pressure ventilation, *PaCO*_*2*_ arterial carbon dioxide tension, *PaO*_*2*_ arterial oxygen tension, *FiO*_*2*_ fraction of inspired oxygen, *ΔPes* inspiratory oesophageal pressure swing, *PTPes* oesophageal pressure–time product, *GCS* Glasgow Coma Scale

^*^Measured over the last 3–5 min of oesophageal pressure recording within 24 h after randomisation

**Fig. 4:**
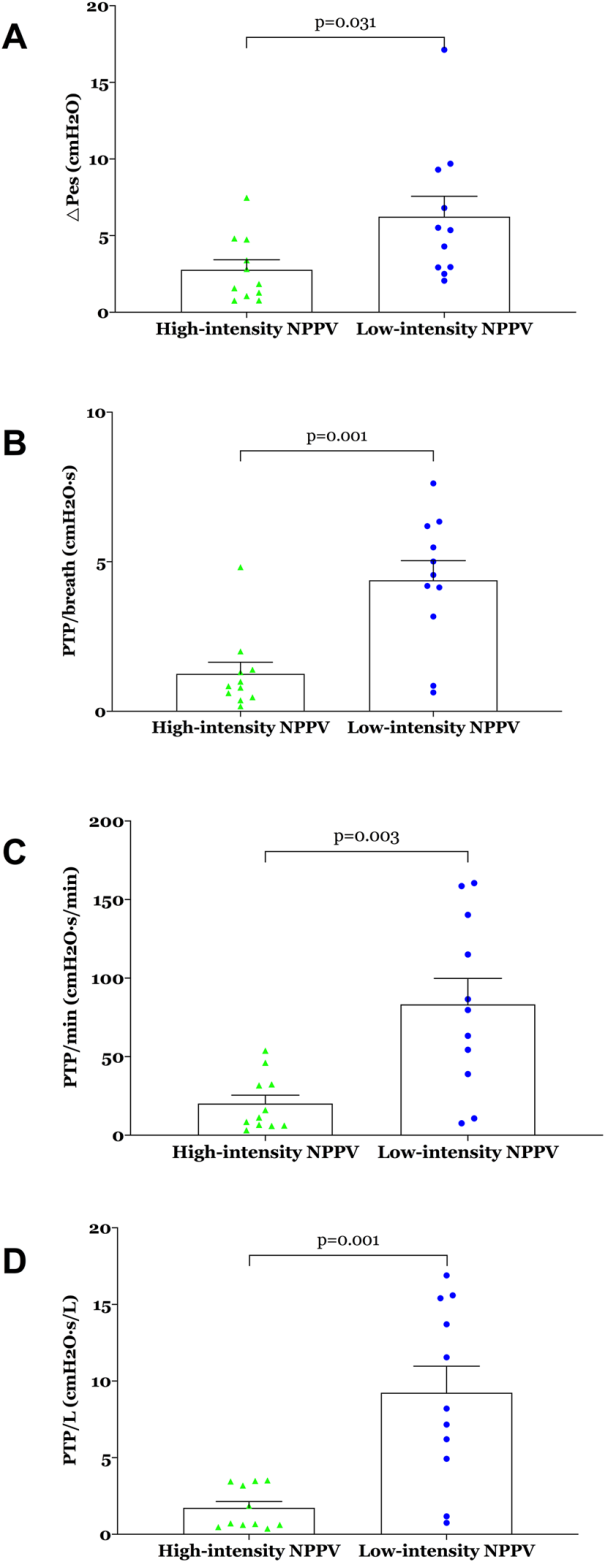
**a** ΔPes, **b** PTPes/breath, **c** PTPes/min, and **d** PTPes/L within 24 h after randomisation. Values are individuals, means, and standard errors. *ΔPes* inspiratory oesophageal pressure swing, *NPPV* noninvasive positive pressure ventilation, *PTPes* oesophageal pressure–time product

During NPPV, ΔPes, PTPes/breath, PTPes/min, and PTPes/L were significantly lower in the high-intensity NPPV group than in the low-intensity NPPV group (Fig. 4; Table 2). Accessory muscle use and the dyspnoea score were significantly lower 24 h after randomisation in the high-intensity NPPV group than in the low-intensity NPPV group (see Table 2 for more details). GCS score, Kelly–Matthay score, and NPPV tolerance score did not differ significantly 24 h after randomisation between the two groups (see Table 2 for more details).

### Patient–ventilator asynchrony, cardiac function, VILI, and adverse events

**Fig. 5:**
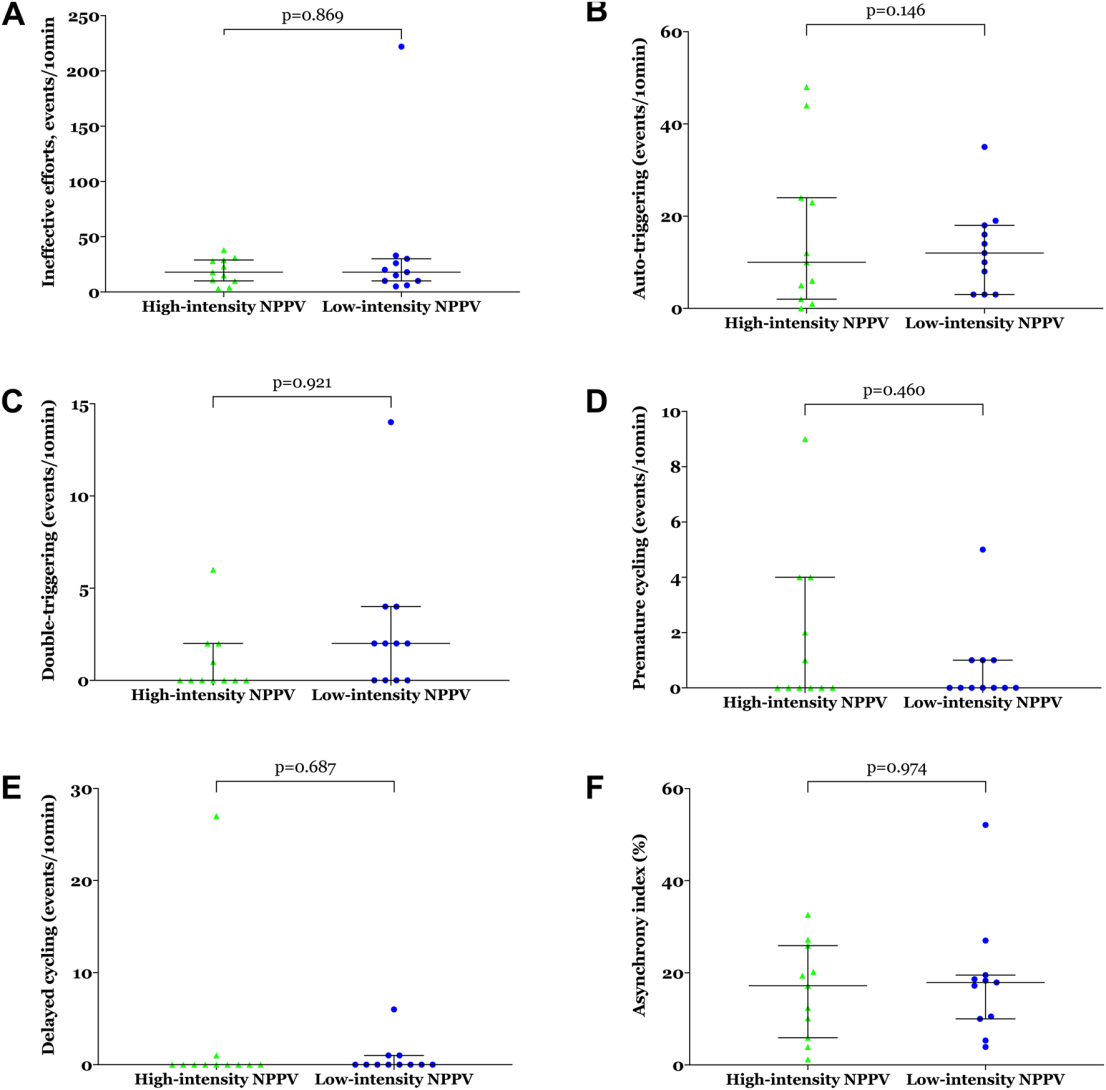
**a** Ineffective efforts, **b** auto-triggering, **c** double-triggering, **d** premature cycling, **e** delayed cycling, and **f** the asynchrony index within 24 h after randomisation. Values are individuals, medians, and interquartile ranges. *NPPV* noninvasive positive pressure ventilation.

**Fig. 6:**
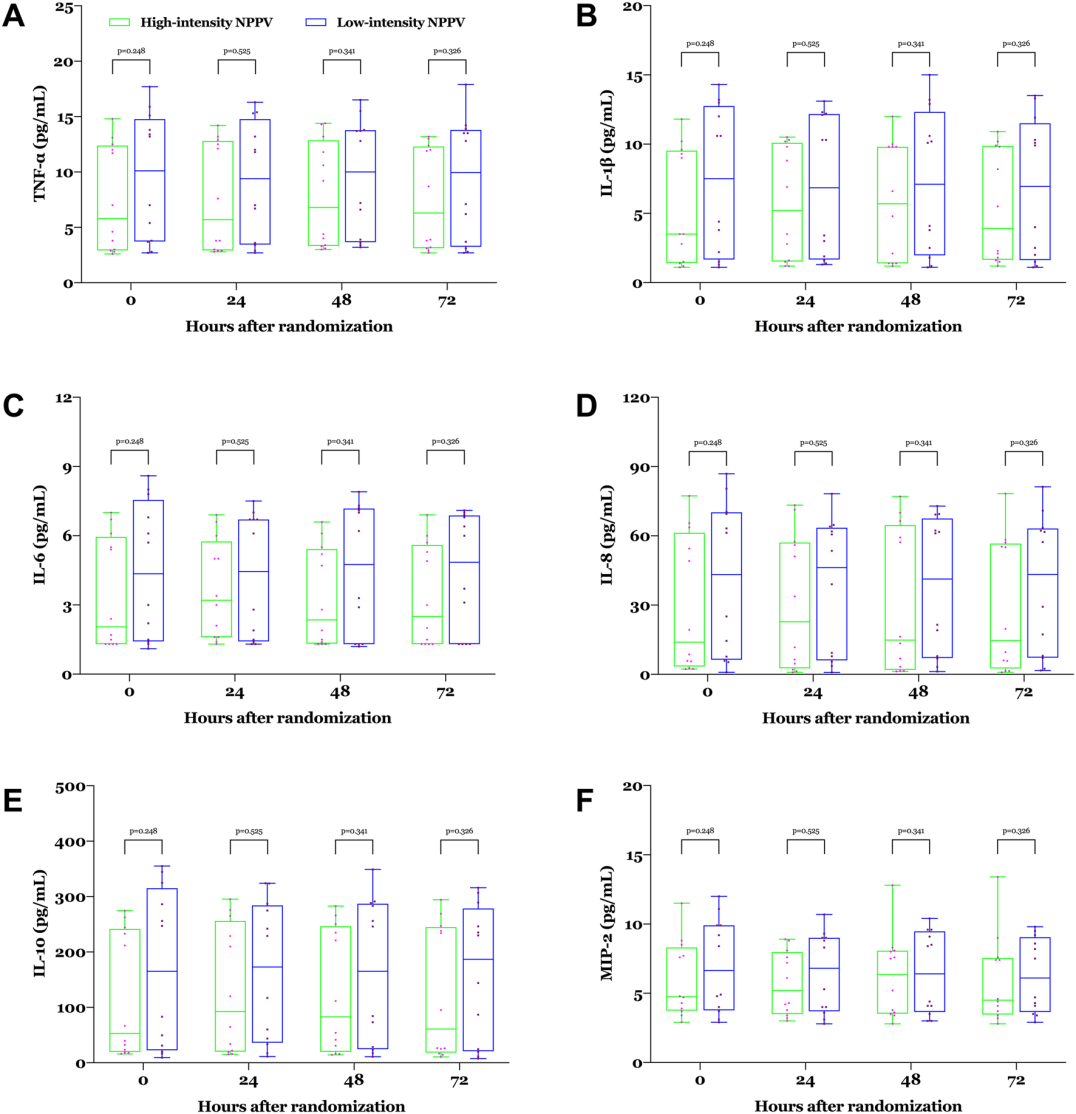
Plasma levels of **a** TNF-α, **b** IL-1β, **c** IL-6, **d** IL-8, **e** IL-10, and **f** MIP-2 from baseline through 72 h after randomisation. Values are individuals, medians, and interquartile ranges with whiskers indicating minimum and maximum. *NPPV* noninvasive positive pressure ventilation, *TNF* tumour necrosis factor, *IL* interleukin, *MIP* macrophage inflammatory protein

During NPPV, no significant between-groups differences were observed in ineffective efforts, auto-triggering, double-triggering, premature cycling, delayed cycling, or the asynchrony index (Fig. 5 and Additional file 1: Table S3). No indicators of cardiac function, including heart rate, blood pressure, N-terminal pro-B-type natriuretic peptide, troponin I, creatine kinase isoenzyme, or echocardiographic findings, differed significantly 24 h after randomisation between the two groups (see Additional file 1: Table S3 for more details). There were no significant between-groups differences in plasma levels of TNF-α, IL-1β, IL-6, IL-8, IL-10, or MIP-2 24 h after randomisation (see Fig. 6 and Additional file 1: Table S3 for more details).

During NPPV, the high-intensity NPPV group did not have a higher incidence of severe NPPV intolerance, but did have a numerically higher incidence of abdominal distention compared to the low-intensity NPPV group (Additional file 1: Table S3). No severe alkalosis occurred, and no patient required arginine.

### Clinical outcomes

One patient in the low-intensity NPPV group required endotracheal intubation, but none in the high-intensity NPPV group did; this between-groups difference was not significant. Similarly, other outcome variables did not differ significantly between the two groups (Fig. S2 and S3 and Additional file 1: Table S4). No complications were observed in either group.

## Discussion

In this physiological RCT involving AECOPD patients in the respiratory ICU, high-intensity NPPV was superior to low-intensity NPPV at decreasing elevated PaCO_2_, reducing inspiratory effort, and alleviating dyspnoea and was similar to low-intensity NPPV at improving consciousness and NPPV tolerance. We found no significant between-groups differences in patient–ventilator asynchrony, cardiac function, VILI, or NPPV-related adverse events.

In line with findings involving stable COPD patients reported by Dreher et al. [15] and Lukácsovits et al. [16], in which high-intensity NPPV reduced PaCO_2_ to a lower level than low-intensity NPPV, we found that high-intensity NPPV resulted in a lower PaCO_2_ than low-intensity NPPV 24 h after randomisation in these AECOPD patients. High-intensity NPPV provides greater pressure support and delivers a higher V_T_ (that averaged above 10 mL/kg of PBW in our trial), augmenting alveolar ventilation and offsetting the extra dead space caused by the face mask, so maximal PaCO_2_ reduction can be achieved only if patients can continuously accept high-intensity NPPV (the patients in our trial did in fact do this). In contrast, low-intensity NPPV provides relatively less pressure support and delivers a smaller V_T_ (that averaged below 10 mL/kg of PBW in our trial); reduced alveolar ventilation cannot be fully augmented, and the decrease in PaCO_2_ is limited. This suggests that high-intensity NPPV is superior to low-intensity NPPV in this regard [1, 6–8]. Moreover, high-intensity NPPV required patients to receive NPPV continuously (but low-intensity NPPV did not), which produced a longer NPPV duration in the high-intensity NPPV group. Thus, the improvement in alveolar ventilation might have been further enhanced and PaCO_2_ might have decreased more markedly. Only four patients in our trial achieved normocapnia, possibly because most patients had end-stage COPD with severe respiratory mechanics and excessive physiologic dead space, so it was difficult to normalise PaCO_2_ despite the use of high-intensity NPPV [20]. This in itself indicates that the use of high-intensity NPPV was more physiologically reasonable for these AECOPD patients than the use of low-intensity NPPV.

ΔPes and PTPes are common measures of inspiratory effort [21]. In our trial, high-intensity NPPV was associated with lower ΔPes and PTPes and produced lower accessory muscle use and dyspnoea scores compared to low-intensity NPPV. This indicates that patients’ inspiratory effort was reduced more with high-intensity NPPV than low-intensity NPPV. In line with our results, Lukácsovits et al. [16] reported that in stable COPD patients, high-intensity NPPV induced a greater reduction in the tidal swing of Pes and transdiaphragmatic pressure and in the pressure–time product of the diaphragm than low-intensity NPPV. Dreher et al. [15] also found that compared to baseline, only high-intensity NPPV (not low-intensity NPPV) resulted in significant improvements in lung function and the Borg dyspnoea scale following walking. This is primarily because noninvasive pressure support during inspiration can rest the respiratory muscle, reduce its effort, and relieve its fatigue, and these effects are more pronounced with higher pressure support [22].

We did not find that high-intensity NPPV helped patients better recover consciousness, mainly as baseline GCS and Kelly–Matthay scores did not worsen substantially in either group. This is similar to the mean GCS score at baseline of 14 reported by Contou et al. [19] and Carrera et al. [23]. It is possible that patients who experience chronic hypercapnic respiratory failure might adapt to the sharp increase in PaCO_2_ [24]. We did not observe between-groups differences in NPPV tolerance or NPPV-related adverse events. One possible explanation for this is that our trial was conducted in a respiratory ICU by staff with considerable experience in NPPV, and all patients received continuous instructions to use NPPV, close monitoring, and standardised NPPV implementation. Possible hyperinflation with a high V_T_ would have worsened patient–ventilator asynchrony (e.g., ineffective efforts) and impeded cardiac function [22, 25, 26]. However, we did not find between-groups differences in any of the measured indicators of patient–ventilator asynchrony or cardiac function. One main reason for this may be that despite increasing V_T_, high-intensity NPPV in our trial did not increase the backup RR, which is different from previously published studies [14, 16], and thus enabled patients to have sufficient expiratory time to achieve lung emptying, without resulting in obvious hyperinflation. We did not find higher plasma concentrations of the measured inflammatory mediators associated with VILI in the high-intensity NPPV group, possibly because we set the safe upper limit for IPAP to 30 cmH_2_O [27, 28], and the actual time for alveolar pressure (one of the key factors leading to VILI) to reach the set maximum pressure might have been shorter than the set inspiratory time because of high airway resistance and circuit leakage [29].

The strengths of this trial include the fact that it is the first RCT involving high-intensity NPPV in AECOPD patients, it used a prospective design and blind randomisation to the assigned strategy, it had a clear protocol for NPPV management and various measurements, and it used multiple variables to investigate the physiological effects of high-intensity NPPV. However, several limitations should be taken into account. First, despite being reasonably estimated and executed according to the plan for this physiological trial, the sample size was not very large, which may have resulted in little power to detect significant between-groups differences, especially in qualitative variables. Second, it is impossible for all investigators and attending physicians in open clinical trials to remain completely blind to treatment group, which may have led to possible bias. Although we defined criteria for all relevant interventions, clinical decisions, and outcome measurements in advance, such bias cannot be entirely controlled. Third, unlike Windisch et al. [14], who described a higher backup RR, we set a lower one, mainly to avoid possible patient–ventilator asynchrony and cardiac dysfunction caused by pulmonary hyperinflation with insufficient expiratory time to achieve lung emptying and because previous findings have shown that the high-pressure component of high-intensity NPPV plays a key role in the management of hypercapnic COPD patients [26]. Fourth, we cannot rule out the possibility that the remarkably prolonged duration of NPPV may have helped decrease elevated PaCO_2_ in the high-intensity NPPV group. Fifth, leakage at the patient–mask interface did not differ significantly between the two groups, and no severe leakage was observed on the basis of intensive monitoring and nursing in our trial. However, high inspiratory pressure might have led to severe leakage in the absence of intensive monitoring and nursing; this, along with proportionally increased intentional leakage through the exhalation port on the mask, might have affected patient–ventilator synchrony and ventilator performance [29, 30]. Finally, because our trial was conducted in a single centre by staff with considerable experience using NPPV to treat AECOPD, caution should be taken when generalising our findings to other settings. Moreover, our findings should not be generalised to patients at high risk for restrictive ventilatory dysfunction and obvious emphysematous bullae, because patients with these conditions were excluded from this trial.

## Conclusions

Our physiological trial indicates that compared to low-intensity NPPV, high-intensity NPPV is more effective at decreasing elevated PaCO_2_, reducing inspiratory effort, and alleviating dyspnoea. Given these physiological findings, an RCT of proper design and power is warranted to determine the clinical efficacy and safety of high-intensity NPPV for treating AECOPD.

## Supplementary Information

**Additional file 1.** Supplementary methods. **Fig. S1**. Representative tracings of the five types of asynchrony. **Fig. S2**. Kaplan–Meier plots of the cumulative incidence of the need for intubation from randomisation to day 28. **Fig. S3**. Kaplan–Meier plots of the cumulative probability of a) remaining on NPPV, b) hospital readmission, and c) survival from randomisation to day 90. **Table S1**. Baseline characteristics of the patients. **Table S2**. NPPV use. **Table S3**. Patient–ventilator asynchrony, cardiac function, ventilator-induced lung injury, and adverse events. **Table S4**. Clinical outcomes.

## Data Availability

The data sets used and/or analysed during the current trial are available from the corresponding author on reasonable request.

## Abbreviations

ABG: Arterial blood gas
AECOPD: Acute exacerbation of chronic obstructive pulmonary disease
COPD: Chronic obstructive pulmonary disease
EPAP: Expiratory positive airway pressure
FEV_1_: Forced expiratory volume in 1 s
FiO_2_: Fraction of inspired oxygen
FVC: Forced vital capacity
GCS: Glasgow Coma Scale
ICU: Intensive care unit
IL: Interleukin
IPAP: Inspiratory positive airway pressure
MIP: Macrophage inflammatory protein
NPPV: Noninvasive positive pressure ventilation
PaCO_2_: Arterial carbon dioxide tension
PaO_2_: Arterial oxygen tension
PBW: Predicted body weight
Pes: Oesophageal pressure
ΔPes: Inspiratory oesophageal pressure swing
PTPes: Oesophageal pressure–time product
RCT: Randomised controlled trial
RR: Respiratory rate
SD: Standard deviation
TNF: Tumour necrosis factor
VILI: Ventilator-induced lung injury
V_T_: Tidal volume
