# Supplementary material for: Physiological effects of high-intensity versus low-intensity noninvasive positive pressure ventilation in patients with acute exacerbation of chronic obstructive pulmonary disease: a randomised controlled trial
Source: Ann Intensive Care. 2022 May 19;12:41. doi: 10.1186/s13613-022-01018-4 (PMC9120318; doi:10.1186/s13613-022-01018-4)
Supplement: Supplementary file 1 — Additional file 1. Supplementary methods. Fig. S1. Representative tracings of the five types of asynchrony. Fig. S2. Kaplan–Meier plots of the cumulative incidence of the need for intubation from randomisation to day 28. Fig. S3. Kaplan–Meier plots of the cumulative probability of a) remaining on NPPV, b) hospital readmission, and c) survival from randomisation to day 90. Table S1. Baseline characteristics of the patients. Table S2. NPPV use. Table S3. Patient–ventilator asynchrony, cardiac function, ventilator-induced lung injury, and adverse events. Table S4. Clinical outcomes. [file 13613_2022_1018_MOESM1_ESM.docx]

**Supplementary methods**

**Exclusion criteria**

Presence of ≥1 of the following:

1. Age <18 years
2. Excessive respiratory secretions with weak cough
3. Obstruction of the upper airway
4. Recent oral, facial, or cranial trauma or surgery
5. Recent gastric or oesophageal surgery
6. High risk for restrictive ventilatory dysfunction (e.g., consolidation or removal of at least one pulmonary lobe, massive pleural effusion, chest wall deformity, continuous strapping with thoracic or abdominal bandage, or severe abdominal distension)
7. Active upper gastrointestinal bleeding
8. Cardiac or respiratory arrest
9. Arterial oxygen tension/fraction of inspired oxygen (PaO_2_/FiO_2_) <100 mmHg
10. Pneumothorax
11. Obvious emphysematous bullae confirmed by computed tomography scan
12. Severe ventricular arrhythmia or myocardial ischemia
13. Severe haemodynamic instability despite fluid repletion and use of vasoactive agents
14. Severe metabolic acidosis
15. Refusal to receive noninvasive positive pressure ventilation (NPPV) or give informed consent
16. Tracheotomy or endotracheal intubation before admission to the intensive care unit
17. A do-not-intubate order

**NPPV withdrawal**

In both groups, provided that clinical conditions were satisfactory and arterial blood gas (ABG) values reached their targets, inspiratory positive airway pressure (IPAP) and daily use of NPPV were gradually decreased until NPPV could be successfully weaned. An attempt to withdraw NPPV was made if daily use was less than 6 h. Weaning was considered successful if patients were able to sustain spontaneous breathing without signs of respiratory distress for 72 h after withdrawal, defined as the presence of at least two of the following: arterial pH <7.35; respiratory rate (RR) >30 breaths/min; PaO_2_ <60 mmHg or oxygen saturation measured by pulse oximetry <90% at FiO_2_ ≥0.4; retraction of the intercostal spaces, use of accessory respiratory muscles, or thoracoabdominal paradoxical movement; or decreased consciousness, agitation, or diaphoresis [1, 2]. In contrast, when patients presented with the aforementioned signs of respiratory distress after withdrawal, weaning was considered to have failed and NPPV was resumed. In the high-intensity NPPV group, weaning was also considered to have failed and NPPV was resumed if arterial carbon dioxide tension (PaCO_2_) increased markedly (i.e., PaCO_2_ should always have been maintained at the targeted level of high-intensity NPPV during the intensive care unit [ICU] stay). If weaning from NPPV had still not been successful by the time patients were discharged from the respiratory ICU, home NPPV with the assigned strategy was recommended.

**Endotracheal intubation**

Endotracheal intubation was considered if either of two criteria was met (arterial pH <7.25 with PaCO_2_ increased by >20% compared to baseline or PaO_2_/FiO_2_ <100 mmHg) and if at least one of the following criteria was met: clinical signs suggestive of severely decreased consciousness (e.g., coma, delirium); excessive respiratory secretions with weak cough; use of accessory respiratory muscles or thoracoabdominal paradoxical movement; severe upper gastrointestinal bleeding with aspiration or vomiting; or severe haemodynamic instability despite fluid repletion and use of vasoactive agents [1, 2]. A crossover to high-intensity NPPV for patients in the low-intensity NPPV group was allowed if these criteria were met. The final decision to intubate was made by the attending physician with the consent of the patient’s next of kin or other surrogate decision makers as appropriate.

**Data collection at baseline**

At baseline, we recorded data regarding demographics; smoking history; lung function (within the previous 1 year or else at ICU discharge); chronic obstructive pulmonary disease-related characteristics; coexisting illness; reason for acute exacerbation; ABGs at ICU admission and at randomisation; and vital signs, laboratory findings, and severity status at randomisation.

**Recording of NPPV parameters**

We recorded NPPV settings (including IPAP, expiratory positive airway pressure [EPAP], and FiO_2_) and the monitored parameters (including tidal volume (V_T_), V_T_/predicted body weight, RR, minute volume, and leakage [at the patient–mask interface]) at baseline and 2, 6, 24, 48, and 72 h after randomisation. Maximal IPAP, maximal EPAP, daily hours of NPPV use over the first 3 days, and total hours and days of NPPV use were also recorded.

**Estimation of inspiratory effort and patient–ventilator asynchrony**

To estimate inspiratory effort and patient–ventilator asynchrony, we placed an oesophageal catheter (CooperSurgical, Trumbull, CT, USA) through the nostril into the oesophagus within 24 h after randomisation. The catheter was initially advanced to a depth of about 55 cm from the nose tip and subsequently withdrawn until the appearance of cardiac artefacts and appropriate negative swings of pressure tracings during spontaneous inspiration to locate the balloon in the lower third of the oesophagus [3, 4]. The balloon was inflated with 0.5 mL air [5].

During NPPV, a pressure transducer (ADInstruments, Bella Vista, New South Wales, Australia) was connected to the oesophageal catheter to record oesophageal pressure (Pes) waveforms continuously for 2 h. At the same time, a pressure transducer (ADInstruments) and a flow transducer (ADInstruments) were placed between the circuit and the mask to record synchronously inspiratory flow and airway pressure waveforms. All transducers were coupled with a dedicated analog-to-digital converter (PowerLab 16/35; ADInstruments) that used corresponding amplifiers of their own (ADInstruments). All signals were amplified, low pass filtered, digitalised at 20 Hz, stored, and analysed with dedicated software (LabChart Pro; ADInstruments).

For inspiratory effort, we measured inspiratory oesophageal pressure swing (ΔPes), oesophageal pressure-time product (PTPes)/breath, PTPes/min, and PTPes/L over the last 3–5 min of Pes recording. ΔPes was defined as the absolute difference between end-expiratory and inspiratory peak Pes in a series of representative breaths divided by the number of breaths (at least 10) [3, 6]. PTPes/breath was defined as the area subtended by the Pes waveform during inspiration in the same series of representative breaths used to measure ΔPes divided by the number of breaths [3]. PTPes/min was defined as the sum of the areas subtended by the Pes waveform during inspiration over the period of 3–5 min divided by the number of minutes [3]. PTPes/L was defined as the sum of the areas subtended by the Pes waveform during inspiration over the same minutes used to measure PTPes/min divided by the sum of minute volume [3, 7].

All asynchrony events (including ineffective efforts, auto-triggering, double-triggering, premature cycling, and delayed cycling) were determined by visual inspection of the tracings of Pes, airway pressure, and flow over the last 10 min of these recordings. Ineffective efforts were identified by a Pes deflection not followed by a ventilator cycle [6]. Auto-triggering was defined as a ventilator cycle without a preceding Pes deflection [8]. Double-triggering was defined as two cycles separated by an expiratory time less than half the average inspiratory time, with the first cycle being triggered by the patient [8]. Premature cycling was defined as an inspiratory time less than half the average inspiratory time [8]. A prolonged cycle was defined as an inspiratory time greater than twice the average inspiratory time [8]. We also computed an asynchrony index, defined as the total number of the above events divided by the total number of ventilator cycles (triggered or not) and of ineffective efforts [8].

**Assessment of accessory muscle use, dyspnoea score, Kelly-Matthay score, and NPPV tolerance**

The use of accessory muscles was assessed according to the following scale: 0, no visible tonic or phasic use of neck muscles; 1, neck muscles taut but with no respiratory modulation (e.g., tonic activity); 2, mild respiratory modulation in neck muscle contraction; 3, moderate phasic activity (no supraclavicular or intercostal indrawing); 4, vigorous phasic activity with indrawing; and 5, vigorous phasic activity with abdominal paradox [9]. Dyspnoea was assessed with a verbal analogue scale, with scores ranging from 0 (no dyspnoea) to 10 (maximum dyspnoea). The Kelly-Matthay score was assessed according to the following scale: 1, alert, follows complex three-step command; 2, alert, follows simple commands; 3, lethargic, but arousable and follows simple commands; 4, stuporous, only intermittently follows simple commands even with vigorous attempts at arousal; 5, comatose, brainstem intact; 6, comatose with brainstem dysfunction [10]. NPPV tolerance was assessed with a verbal analogue scale with scores ranging from 0 (severe intolerance with the need for suspension of NPPV) to 4 (maximum comfort) [11].

**Measurement of inflammatory mediators**

Patients’ venous blood was taken in tubes containing ethylenediaminetetraacetic acid. The blood samples were immediately centrifuged at 3,000 rpm for 10 min. Extract supernatant as a plasma sample was collected and stored at –80°C until detection. A single technician used commercially available enzyme-linked immunosorbent assay kits (Jianglai Bio, Shanghai, China) to measure concentrations of mediators according to the manufacturer’s instructions.

**Recording of adverse events and patient outcomes**

We recorded whether adverse events (e.g., severe NPPV intolerance, severe leakage [>25 L/min], nasal/facial skin necrosis, conjunctivitis, sinus/ear pain, nasal/oral dryness, abdominal distention, aspiration, hypotension, acute respiratory distress syndrome, pneumothorax, or claustrophobia) occurred during the ICU stay and measured abdominal circumference at navel level at baseline and 2, 6, 24, 28, and 72 h after randomisation.

Concerning patient outcomes, we recorded the need for intubation, successful weaning of NPPV, mortality (in the ICU, at day 28, and at day 90), live ICU discharge, live ICU discharge within 28 days after randomisation, ICU length of stay, ICU length of stay after randomisation, ICU-free days within 28 days after randomisation, ICU costs, suggestion for home NPPV with ICU discharge, and 90-day home NPPV use and hospital readmission. We also observed whether any complications (e.g., nosocomial pneumonia, sepsis or septic shock, multiple organ failure, acute myocardial infarction, life-threatening ventricular arrhythmia, cardiogenic shock, gastrointestinal bleeding, pulmonary embolism, or severe alkalosis) occurred during the ICU stay. Patients were followed for 90 days after randomisation.

**Statistical analysis**

Kaplan–Meier curves were used to determine the cumulative incidence of intubation within 28 days after randomisation and the cumulative probability of remaining on NPPV, hospital readmission, and survival within 90 days after randomisation; the curves of the two groups were compared with the log-rank test (Figs. S2, S3). Abdominal circumference was compared within the same group using analysis of variance for repeated measures, and Bonferroni’s adjustment for multiplicity of tests was applied for post hoc comparisons to ensure that the total error rate did not exceed 0.05 (Table S3). Differences with p < 0.05 were considered statistically significant, except for those from multiple comparisons.

**References**

1. Cao Z, Luo Z, Hou A, Nie Q, Xie B, An X, et al. Volume-targeted versus pressure-limited noninvasive ventilation in subjects with acute hypercapnic respiratory failure: a multicenter randomized controlled trial. Respir Care. 2016;61:1440-50.
2. Luo Z, Wu C, Li Q, Zhu J, Pang B, Shi Y, et al. High-intensity versus low-intensity noninvasive positive pressure ventilation in patients with acute exacerbation of chronic obstructive pulmonary disease (HAPPEN): study protocol for a multicenter randomized controlled trial. Trials. 2018;19:645.
3. Mauri T, Turrini C, Eronia N, Grasselli G, Volta CA, Bellani G, et al. Physiologic effects of high-flow nasal cannula in acute hypoxemic respiratory failure. Am J Respir Crit Care Med. 2017;195:1207-15.
4. Mauri T, Yoshida T, Bellani G, Goligher EC, Carteaux G, Rittayamai N, et al. Esophageal and transpulmonary pressure in the clinical setting: meaning, usefulness and perspectives. Intensive Care Med. 2016;42:1360-73.
5. Akoumianaki E, Maggiore SM, Valenza F, Bellani G, Jubran A, Loring SH, et al. The application of esophageal pressure measurement in patients with respiratory failure. Am J Respir Crit Care Med. 2014;189:520-31.
6. Fraticelli AT, Lellouche F, L’Her E, Taille S, Mancebo J, Brochard L. Physiological effects of different interfaces during noninvasive ventilation for acute respiratory failure. Crit Care Med. 2009;37:939-45.
7. Costa R, Navalesi P, Antonelli M, Cavaliere F, Craba A, Proietti R, et al. Physiologic evaluation of different levels of assistance during noninvasive ventilation delivered through a helmet. Chest. 2005;128:2984-90.
8. Thille AW, Rodriguez P, Cabello B, Lellouche F, Brochard L. Patient-ventilator asynchrony during assisted mechanical ventilation. Intensive Care Med. 2006;32:1515-22.
9. Patrick W, Webster K, Ludwig L, Roberts D, Wiebe P, Younes M. Noninvasive positive-pressure ventilation in acute respiratory distress without prior chronic respiratory failure. Am J Respir Crit Care Med. 1996;153:1005-11.
10. Scala R, Naldi M, Archinucci I, Coniglio G, Nava S. Noninvasive positive pressure ventilation in patients with acute exacerbations of COPD and varying levels of consciousness. Chest. 2005;128:1657-66.
11. Thille AW, Contou D, Fragnoli C, Cordoba-Izquierdo A, Boissier F, Brun-Buisson C. Non-invasive ventilation for acute hypoxemic respiratory failure: intubation rate and risk factors. Crit Care. 2013;17:R269.

**Fig. S1 Representative tracings of the five types of asynchrony.** Pes oesophageal pressure.

**
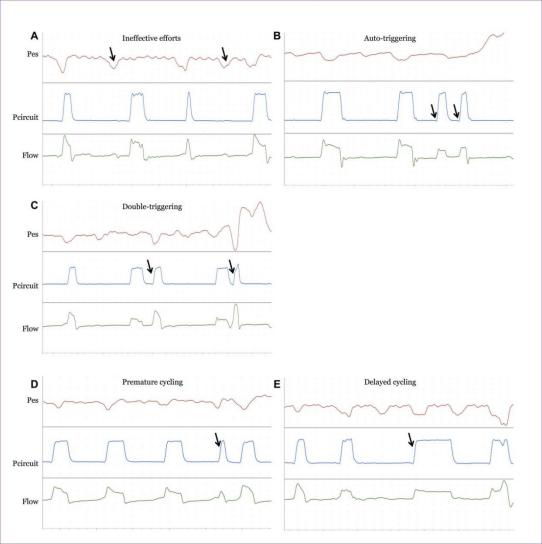
**

**Fig. S2 Kaplan–Meier plots of the cumulative incidence of the need for intubation from randomisation to day 28.** NPPV noninvasive positive pressure ventilation.

**
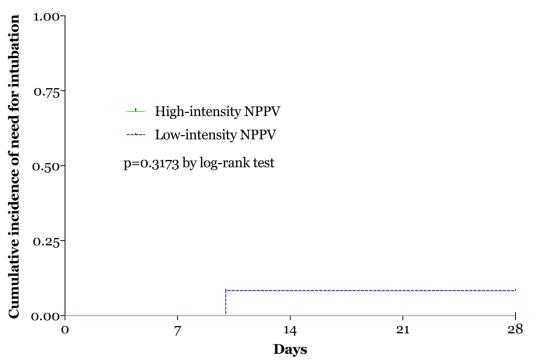
**

**Fig. S3 Kaplan–Meier plots of the cumulative probability of a) remaining on NPPV, b) hospital readmission, and c) survival from randomisation to day 90.** NPPV noninvasive positive pressure ventilation.

**
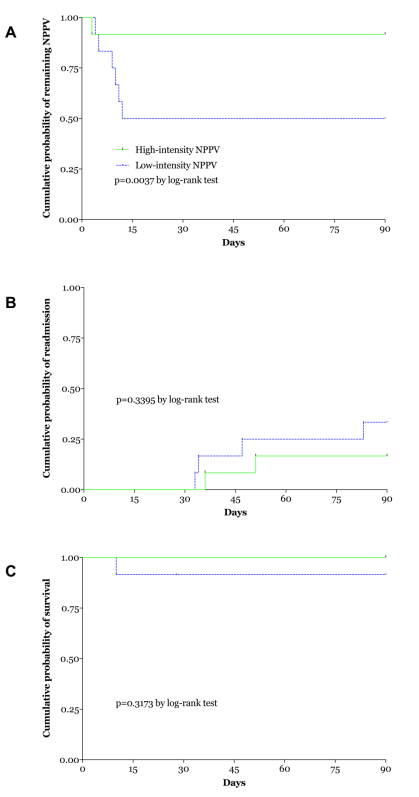
**

| **Table S1 Baseline characteristics of the patients** | | | | |
| --- | --- | --- | --- | --- |
| Characteristic | | High-intensity NPPV (n = 12) | Low-intensity NPPV (n = 12) | p value |
| COPD-related baseline characteristics | | | | |
|  | Disease course, year | 10.0 (10.0–20.0) | 17.5 (10.0–24.5) | 0.227 |
|  | GOLD class IV, n (%) | 11 (91.7) | 9 (75.0) | 0.590 |
|  | GOLD class III, n (%) | 1 (8.3) | 3 (25.0) | 0.590 |
|  | mMRC score | 4.0 (4.0–4.0) | 4.0 (3.0–4.0) | 0.131 |
|  | CAT score | 31.2 ± 4.6 | 29.2 ± 5.9 | 0.385 |
|  | Long-term inhaled bronchodilators, n (%) | 11 (91.7) | 10 (83.3) | >0.999 |
|  | Duration of bronchodilator use, year | 5.9 ± 4.6 | 5.3 ± 4.5 | 0.723 |
|  | Long-term inhaled corticosteroids, n (%) | 11 (91.7) | 10 (83.3) | >0.999 |
|  | Duration of corticosteroid use, year | 5.1 ± 3.3 | 5.3 ± 4.5 | 0.919 |
|  | Long-term oxygen therapy, n (%) | 12 (100.0) | 10 (83.3) | 0.478 |
|  | Duration of oxygen therapy, year | 7.0 (2.5–10.0) | 2.0 (1.0–7.5) | 0.109 |
|  | Long-term NPPV, n (%) | 6 (50.0) | 7 (58.3) | >0.999 |
|  | Duration of NPPV, year | 1.0 (0.0–5.5) | 1.0 (0.0–3.8) | 0.903 |
|  | Hospitalisation within previous 1 year, n (%) | 9 (75.0) | 11 (91.7) | 0.590 |
|  | Hospitalisation frequency, n/year | 1.0 (0.25–1.0) | 1.5 (1.0–2.0) | 0.061 |
|  | ICU admission within previous 1 year, n (%) | 4 (33.3) | 3 (25.0) | >0.999 |
|  | ICU admission frequency, n/year | 0.0 (0.0–1.0) | 0.0 (0.0–0.8) | 0.771 |
| Coexisting illness | | | | |
|  | Hypertensive heart disease, n (%) | 5 (41.7) | 6 (50.0) | >0.999 |
|  | Ischemic heart disease, n (%) | 4 (33.3) | 4 (33.3) | >0.999 |
|  | Prior myocardial infarction, n (%) | 2 (16.7) | 1 (8.3) | >0.999 |
|  | Prior percutaneous coronary intervention, n (%) | 2 (16.7) | 1 (8.3) | >0.999 |
|  | Atrial fibrillation, n (%) | 1 (8.3) | 4 (33.3) | 0.317 |
|  | Diabetes mellitus, n (%) | 4 (33.3) | 3 (25.0) | >0.999 |
|  | Chronic heart failure, n (%) | 2 (16.7) | 3 (25.0) | >0.999 |
|  | Chronic renal failure, n (%) | 1 (8.3) | 1 (8.3) | >0.999 |
|  | Cerebrovascular disease, n (%) | 1 (8.3) | 2 (16.7) | >0.999 |
|  | Solid cancer, n (%) | 3 (25.0) | 3 (25.0) | >0.999 |
| Vital signs at randomisation | | | | |
|  | Body temperature, °C | 36.5 ± 0.4 | 36.4 ± 0.3 | 0.433 |
|  | RR, breaths/min | 21.8 ± 6.8 | 21.9 ± 2.7 | 0.969 |
|  | SpO_2_, % | 95.6 ± 3.2 | 95.2 ± 2.8 | 0.638 |
|  | Heart rate, beats/min | 82.6 ± 12.9 | 86.3 ± 23.2 | 0.637 |
|  | Systolic blood pressure, mmHg | 121.9 ± 17.8 | 121.8 ± 12.0 | 0.979 |
|  | Diastolic blood pressure, mmHg | 68.8 ± 9.7 | 68.1 ± 8.3 | 0.841 |
| Laboratory findings at randomisation | | | | |
|  | Leukocyte count, ×10^9^/L | 7.6 ± 3.1 | 9.1 ± 2.6 | 0.199 |
|  | Neutrophil count, ×10^9^/L | 5.7 ± 2.1 | 7.4 ± 3.1 | 0.130 |
|  | Haemoglobin, g/L | 120.4 ± 35.2 | 129.1 ± 26.0 | 0.500 |
|  | Platelet count, ×10^9^/L | 177.5 ± 67.8 | 226.9 ± 63.8 | 0.080 |
|  | Alanine aminotransferase, U/L | 20.5 (11.1–23.4) | 17.7 (13.3–56.0) | 0.603 |
|  | Aspartate transaminase, U/L | 21.3 (14.6–24.4) | 19.8 (12.3–28.5) | 0.564 |
|  | Total bilirubin, ummol/L | 10.5 (6.9–18.0) | 9.5 (7.8–10.8) | 0.686 |
|  | Direct bilirubin, ummol/L | 3.2 (1.6–3.9) | 1.9 (1.6–3.1) | 0.297 |
|  | Albumin, g/L | 31.0 ± 5.8 | 31.7 ± 5.6 | 0.760 |
|  | Urea nitrogen, mmol/L | 6.4 (4.3–16.6) | 8.5 (5.2–9.5) | 0.603 |
|  | Creatinine, umol/L | 55.5 (46.0–176.7) | 68.2 (45.3–103.2) | 0.817 |
|  | Potassium, mmol/L | 4.4 ± 0.6 | 4.3 ± 0.7 | 0.639 |
|  | Sodium, mmol/L | 137.0 ± 5.1 | 140.5 ± 3.5 | 0.063 |
|  | Chlorine, mmol/L | 92.7 ± 6.0 | 95.9 ± 7.5 | 0.272 |
|  | D-dimer, mg/L | 0.21 (0.09–0.28) | 0.19 (0.14–0.26) | 0.817 |
|  | Prothrombin time, s | 12.3 (11.0–14.0) | 11.6 (11.1–14.0) | 0.686 |
|  | Prothrombin activity, % | 89.4 ± 18.7 | 91.0 ± 17.5 | 0.832 |
|  | Activated partial thromboplastin time, s | 31.8 (29.3–36.0) | 31.0 (30.0–33.0) | >0.999 |
|  | Fibrinogen, g/L | 2.9 ± 0.8 | 3.4 ± 0.8 | 0.126 |
|  | N-terminal pro B-type natriuretic peptide, pg/mL | 751 (145–2789) | 1131 (127–2130) | 0.908 |
|  | Troponin I, ng/mL | 0.04 ± 0.02 | 0.04 ± 0.01 | 0.444 |
|  | Creatine kinase isoenzyme, ng/mL | 14.0 ± 7.2 | 15.5 ± 4.8 | 0.537 |
| Severity status at randomisation | | | | |
|  | APACHE II score | 17.6 ± 4.9 | 15.4 ± 3.6 | 0.231 |
|  | SAPS II score | 32.7 ± 12.0 | 30.0 ± 6.9 | 0.497 |
| Data are presented as means ± standard deviations, medians (25th–75th percentiles), or frequencies (percentages) of patients as appropriate  NPPV noninvasive positive pressure ventilation, COPD chronic obstructive pulmonary disease, GOLD Global Initiative for Chronic Obstructive Lung Disease, mMRC modified Medical Research Council, CAT COPD Assessment Test, ICU intensive care unit, RR respiratory rate, SpO_2_ oxygen saturation measured by pulse oximetry, APACHE Acute Physiology and Chronic Health Evaluation, SAPS Simplified Acute Physiology Score | | | | |

| **Table S2 NPPV use** | | | | | |
| --- | --- | --- | --- | --- | --- |
| Variable | | | High-intensity NPPV  (n = 12) | Low-intensity NPPV  (n = 12) | p value |
| Setting | | |  |  |  |
|  | IPAP, cmH_2_O | |  |  |  |
|  |  | Baseline | 16.0 (15.3–17.8) | 15.5 (14.3–16.0) | 0.114 |
|  |  | 2 h | 26.0 (25.3–28.0) | 15.5 (15.0–17.5) | 0.000 |
|  |  | 6 h | 26.0 (26.0–28.0) | 15.5 (15.0–18.0) | 0.000 |
|  |  | 24 h | 28.0 (26.0–28.0) | 15.5 (15.0–17.5) | 0.000 |
|  |  | 48 h | 28.0 (28.0–28.0) | 15.0 (15.0–17.5) | 0.000 |
|  |  | 72 h | 28.0 (28.0–28.0) | 15.0 (15.0–17.5) | 0.000 |
|  | EPAP, cmH_2_O | |  |  |  |
|  |  | Baseline | 6.0 (5.0–6.0) | 5.0 (5.0–6.0) | 0.333 |
|  |  | 2 h | 6.0 (6.0–6.0) | 5.0 (5.0–6.0) | 0.059 |
|  |  | 6 h | 6.0 (5.3–6.0) | 5.0 (5.0–6.0) | 0.115 |
|  |  | 24 h | 6.0 (5.0–6.0) | 5.0 (4.3–6.0) | 0.106 |
|  |  | 48 h | 6.0 (5.0–6.0) | 5.0 (5.0–6.0) | 0.140 |
|  |  | 72 h | 6.0 (5.0–6.0) | 5.0 (5.0–6.0) | 0.140 |
|  | FiO_2_, % | |  |  |  |
|  |  | Baseline | 35.0 (35.0–40.0) | 37.5 (31.3–40.0) | 0.904 |
|  |  | 2 h | 35.0 (30.0–38.8) | 35.0 (30.0–40.0) | 0.976 |
|  |  | 6 h | 35.0 (30.0–35.0) | 35.0 (30.0–38.8) | 0.557 |
|  |  | 24 h | 35.0 (30.0–35.0) | 35.0 (30.0–35.0) | 0.925 |
|  |  | 48 h | 30.0 (30.0–35.0) | 30.0 (30.0–35.0) | 0.827 |
|  |  | 72 h | 30.0 (30.0–35.0) | 30.0 (30.0–35.0) | 0.851 |
| Monitoring | | |  |  |  |
|  | RR, breaths/min | |  |  |  |
|  |  | Baseline | 21.8 ± 6.8 | 21.9 ± 2.7 | 0.969 |
|  |  | 2 h | 16.3 ± 3.6 | 19.3 ± 2.9 | 0.035 |
|  |  | 6 h | 15.8 ± 2.9 | 19.5 ± 4.0 | 0.017 |
|  |  | 24 h | 16.4 ± 3.4 | 20.8 ± 4.6 | 0.015 |
|  |  | 48 h | 15.8 ± 3.0 | 19.2 ± 4.3 | 0.038 |
|  |  | 72 h | 16.0 ± 3.3 | 19.4 ± 4.2 | 0.037 |
|  | V_T_, mL | |  |  |  |
|  |  | Baseline | 407 ± 77 | 447 ± 64 | 1.834 |
|  |  | 2 h | 686 ± 127 | 460 ± 52 | 0.000 |
|  |  | 6 h | 713 ± 132 | 481 ± 58 | 0.000 |
|  |  | 24 h | 734 ± 159 | 505 ± 61 | 0.000 |
|  |  | 48 h | 752 ± 139 | 501 ± 74 | 0.000 |
|  |  | 72 h | 834 ± 141 | 516 ± 87 | 0.000 |
|  | V_T_/PBW, mL/kg | |  |  |  |
|  |  | Baseline | 6.6 ± 1.1 | 7.3 ± 1.3 | 0.130 |
|  |  | 2 h | 11.1 ± 1.8 | 7.6 ± 1.4 | 0.000 |
|  |  | 6 h | 11.5 ± 1.7 | 7.9 ± 1.3 | 0.000 |
|  |  | 24 h | 11.8 ± 2.2 | 8.3 ± 1.3 | 0.000 |
|  |  | 48 h | 12.1 ± 1.9 | 8.2 ± 1.4 | 0.000 |
|  |  | 72 h | 13.5 ± 2.1 | 8.4 ± 1.4 | 0.000 |
|  | V_m_, L/min | |  |  |  |
|  |  | Baseline | 9.0 ± 2.1 | 9.4 ± 2.1 | 0.628 |
|  |  | 2 h | 11.4 ± 2.8 | 9.1 ± 1.0 | 0.019 |
|  |  | 6 h | 11.7 ± 2.2 | 9.4 ± 1.8 | 0.011 |
|  |  | 24 h | 12.4 ± 2.5 | 10.3 ± 1.7 | 0.025 |
|  |  | 48 h | 12.1 ± 2.7 | 9.5 ± 2.0 | 0.014 |
|  |  | 72 h | 13.5 ± 2.6 | 10.3 ± 2.6 | 0.007 |
|  | Leakage, L/min | |  |  |  |
|  |  | Baseline | 5.0 (0.0–11.8) | 6.0 (5.0–10.0) | 0.519 |
|  |  | 2 h | 10.0 (5.8–10.8) | 10.0 (5.0–10.0) | 0.789 |
|  |  | 6 h | 10.0 (6.3–16.5) | 9.0 (5.0–10.0) | 0.202 |
|  |  | 24 h | 10.0 (8.9–13.5) | 10.0 (6.0–13.8) | 0.539 |
|  |  | 48 h | 10.5 (10.0–14.5) | 10.0 (8.3–10.0) | 0.060 |
|  |  | 72 h | 10.0 (10.0–20.0) | 10.0 (5.8–14.8) | 0.255 |
| Recording | | |  |  |  |
|  | Maximal IPAP, cmH_2_O | | 28.0 (28.0–29.5) | 16.0 (15.0–18.0) | 0.000 |
|  | Maximal EPAP, cmH_2_O | | 6.0 (6.0–6.0) | 5.0 (5.0–6.0) | 0.074 |
|  | Daily hours of NPPV, h | |  |  |  |
|  |  | Day 1 | 21.8 ± 2.1 | 15.3 ± 4.7 | 0.001 |
|  |  | Day 2 | 21.6 ± 2.4 | 13.5 ± 5.7 | 0.000 |
|  |  | Day 3 | 21.4 ± 2.0 | 13.5 ± 6.0 | 0.001 |
|  | Total hours of NPPV, h | | 129 (91–209) | 68 (49–187) | 0.073 |
|  | Total days of NPPV, day | | 7.5 (5.0–11.8) | 8.5 (5.5–10.8) | 0.772 |
| Data are presented as means ± standard deviations, medians (25th–75th percentiles), or frequencies (percentages) of patients as appropriate  NPPV noninvasive positive pressure ventilation, IPAP inspiratory positive airway pressure, EPAP expiratory positive airway pressure, FiO_2_ fraction of inspired oxygen, RR respiratory rate, V_T_ tidal volume, PBW predicted body weight, V_m_ minute volume | | | | | |

| **Table S3 Patient–ventilator asynchrony, cardiac function, ventilator-induced lung injury, and adverse events** | | | | | |
| --- | --- | --- | --- | --- | --- |
| Variable | | | High-intensity NPPV  (n = 12) | Low-intensity NPPV  (n = 12) | p value |
| Patient–ventilator asynchrony (n = 22, 11, 11)* | | | | |  |
|  | Ineffective efforts, events/10 min | | 18.0 (10.0–29.0) | 18.0 (10.0–30.0) | 0.869 |
|  | Auto-triggering, events/10 min | | 10.0 (2.0–24.0) | 12.0 (3.0–18.0) | 0.146 |
|  | Double-triggering, events/10 min | | 0.0 (0.0–2.0) | 2.0 (0.0–4.0) | 0.921 |
|  | Premature cycling, events/10 min | | 0.0 (0.0–4.0) | 0.0 (0.0–1.0) | 0.460 |
|  | Delayed cycling, events/10 min | | 0.0 (0.0–0.0) | 0.0 (0.0–1.0) | 0.687 |
|  | Asynchrony index, % | | 17.2 (5.9–25.9) | 17.9 (10.0–19.5) | 0.974 |
| Cardiac function | | | | |  |
|  | Heart rate, beats/min | |  |  |  |
|  |  | Baseline | 82.3 ± 12.9 | 86.3 ± 23.1 | 0.637 |
|  |  | 2 h | 78.9 ± 15.2 | 79.4 ± 22.3 | 0.949 |
|  |  | 6 h | 76.4 ± 12.9 | 82.9 ± 19.7 | 0.349 |
|  |  | 24 h | 79.8 ± 13.8 | 85.1 ± 21.4 | 0.350 |
|  |  | 48 h | 77.3 ± 12.7 | 77.2 ± 17.0 | 0.979 |
|  |  | 72 h | 76.6 ± 13.3 | 79.3 ± 17.6 | 0.679 |
|  | Systolic blood pressure, mmHg | |  |  |  |
|  |  | Baseline | 121.9 ± 17.8 | 121.8 ± 12.0 | 0.979 |
|  |  | 2 h | 120.5 ± 16.2 | 122.2 ± 16.6 | 0.806 |
|  |  | 6 h | 120.3 ± 18.7 | 124.7 ± 11.6 | 0.494 |
|  |  | 24 h | 121.6 ± 13.6 | 122.6 ± 14.9 | 0.865 |
|  |  | 48 h | 118.8 ± 15.0 | 127.9 ± 7.6 | 0.075 |
|  |  | 72 h | 121.6 ± 17.6 | 126.8 ± 16.1 | 0.461 |
|  | Diastolic blood pressure, mmHg | |  |  |  |
|  |  | Baseline | 68.8 ± 9.7 | 68.1 ± 8.3 | 0.841 |
|  |  | 2 h | 67.2 ± 11.6 | 68.9 ± 12.6 | 0.727 |
|  |  | 6 h | 64.0 ± 12.3 | 71.0 ± 9.5 | 0.133 |
|  |  | 24 h | 68.3 ± 7.8 | 69.8 ± 13.8 | 0.734 |
|  |  | 48 h | 65.1 ± 10.7 | 67.7 ± 13.0 | 0.601 |
|  |  | 72 h | 65.3 ± 7.8 | 69.8 ± 15.8 | 0.387 |
|  | N-terminal pro-B-type natriuretic peptide, pg/mL | | | |  |
|  |  | Baseline | 751 (145–2789) | 1131 (127–2130) | 0.908 |
|  |  | 24 h | 540 (92–1704) | 781 (135–1356) | 0.686 |
|  |  | 48 h | 357 (67–1608) | 792 (300–1200) | 0.326 |
|  |  | 72 h | 601 (75–2115) | 760 (116–1989) | 0.773 |
|  | Troponin I, ng/mL | |  |  |  |
|  |  | Baseline | 0.04 ± 0.02 | 0.04 ± 0.01 | 0.444 |
|  |  | 24 h | 0.05 ± 0.02 | 0.04 ± 0.02 | 0.360 |
|  |  | 48 h | 0.04 ± 0.02 | 0.04 ± 0.02 | 0.709 |
|  |  | 72 h | 0.04 ± 0.02 | 0.03 ± 0.01 | 0.293 |
|  | Creatine kinase isoenzyme, ng/mL | | | |  |
|  |  | Baseline | 14.0 ± 7.2 | 15.5 ± 4.8 | 0.537 |
|  |  | 24 h | 15.5 ± 5.6 | 15.3 ± 5.9 | 0.934 |
|  |  | 48 h | 15.6 ± 7.2 | 17.3 ± 9.3 | 0.624 |
|  |  | 72 h | 15.8 ± 6.6 | 13.1 ± 5.0 | 0.271 |
|  | Left ventricular ejection fraction, % | |  |  |  |
|  |  | Baseline | 67.3 ± 3.7 | 65.5 ± 7.1 | 0.458 |
|  |  | 24 h | 67.4 ± 4.0 | 64.1 ± 5.1 | 0.089 |
|  |  | 48 h | 64.8 ± 5.8 | 64.9 ± 7.0 | 0.950 |
|  |  | 72 h | 66.2 ± 4.9 | 63.2 ± 9.3 | 0.333 |
|  | Left ventricular end-diastolic diameter, mm | | | |  |
|  |  | Baseline | 45.8 ± 4.8 | 46.0 ± 7.3 | 0.922 |
|  |  | 24 h | 44.3 ± 4.4 | 46.0 ± 3.4 | 0.311 |
|  |  | 48 h | 43.9 ± 5.2 | 46.7 ± 5.4 | 0.231 |
|  |  | 72 h | 43.7 ± 4.2 | 46.9 ± 3.6 | 0.053 |
|  | Left ventricular end-systolic diameter, mm | | | |  |
|  |  | Baseline | 29.2 ± 4.0 | 30.2 ± 6.2 | 0.644 |
|  |  | 24 h | 27.7 ± 7.7 | 29.8 ± 3.1 | 0.083 |
|  |  | 48 h | 29.4 ± 4.4 | 30.3 ± 5.5 | 0.659 |
|  |  | 72 h | 28.2 ± 3.8 | 30.7 ± 3.9 | 0.126 |
|  | Right atrium longitudinal diameter, mm | | | |  |
|  |  | Baseline | 48.1 ± 4.5 | 51.8 ± 6.2 | 0.112 |
|  |  | 24 h | 46.3 ± 5.9 | 50.3 ± 6.4 | 0.133 |
|  |  | 48 h | 45.5 ± 5.6 | 51.1 ± 8.1 | 0.062 |
|  |  | 72 h | 46.0 ± 5.1 | 50.6 ± 7.3 | 0.089 |
|  | Right atrium transverse diameter, mm | | | |  |
|  |  | Baseline | 34.8 ± 3.3 | 37.7 ± 4.0 | 0.062 |
|  |  | 24 h | 33.0 ± 5.0 | 36.0 ± 3.7 | 0.112 |
|  |  | 48 h | 35.8 ± 4.6 | 37.6 ± 7.1 | 0.480 |
|  |  | 72 h | 33.8 ± 6.3 | 36.8 ± 5.0 | 0.222 |
|  | Pulmonary artery pressure, mm | | | |  |
|  |  | Baseline | 39.9 ± 11.2 | 46.1 ± 16.8 | 0.302 |
|  |  | 24 h | 38.1 ± 8.1 | 40.4 ± 11.8 | 0.577 |
|  |  | 48 h | 36.2 ± 8.0 | 39.8 ± 12.4 | 0.409 |
|  |  | 72 h | 32.5 ± 9.6 | 41.5 ± 12.1 | 0.057 |
|  | Maximal mitral E wave velocity, cm/s | |  |  |  |
|  |  | Baseline | 73.8 ± 23.6 | 91.0 ± 16.6 | 0.052 |
|  |  | 24 h | 73.8 ± 19.4 | 88.3 ± 25.7 | 0.135 |
|  |  | 48 h | 73.1 ± 26.1 | 81.5 ± 29.9 | 0.470 |
|  |  | 72 h | 69.3 ± 24.7 | 80.5 ± 14.6 | 0.191 |
|  | Maximal mitral A wave velocity, cm/s | |  |  |  |
|  |  | Baseline | 87.6 ± 27.2 | 97.8 ± 29.7 | 0.387 |
|  |  | 24 h | 86.4 ± 17.6 | 88.1 ± 21.9 | 0.839 |
|  |  | 48 h | 90.9 ± 23.0 | 94.3 ± 26.2 | 0.737 |
|  |  | 72 h | 92.5 ± 26.0 | 92.8 ± 22.8 | 0.974 |
|  | Ratio of maximal mitral E wave and A wave velocities | | | |  |
|  |  | Baseline | 0.8 (0.6–1.2) | 0.9 (0.7–1.1) | 0.419 |
|  |  | 24 h | 0.8 (0.7–1.1) | 0.9 (0.7–1.2) | 0.299 |
|  |  | 48 h | 0.7 (0.6–1.0) | 0.8 (0.7–1.2) | 0.386 |
|  |  | 72 h | 0.8 (0.6–0.8) | 0.8 (0.7–1.1) | 0.299 |
| Ventilator-induced lung injury | | | | |  |
|  | TNF-α, pg/mL | |  |  |  |
|  |  | Baseline | 5.8 (2.9–12.4) | 10.1 (3.7–14.8) | 0.248 |
|  |  | 24 h | 5.7 (2.9–12.8) | 9.4 (3.4–14.8) | 0.525 |
|  |  | 48 h | 6.8 (3.3–12.9) | 10.0 (3.6–13.8) | 0.341 |
|  |  | 72 h | 6.3 (3.1–12.3) | 9.9 (3.2–13.8) | 0.326 |
|  | IL-1β, pg/mL | |  |  |  |
|  |  | Baseline | 3.5 (1.4–9.5) | 7.5 (1.6–12.7) | 0.214 |
|  |  | 24 h | 5.2 (1.5–10.1) | 6.9 (1.6–12.2) | 0.312 |
|  |  | 48 h | 5.7 (1.4–9.8) | 7.1 (2.0–12.3) | 0.326 |
|  |  | 72 h | 3.9 (1.6–9.8) | 6.9 (1.6–11.5) | 0.436 |
|  | IL-6, pg/mL | |  |  |  |
|  |  | Baseline | 2.0 (1.3–6.0) | 2.0 (1.3–6.0) | 0.371 |
|  |  | 24 h | 3.2 (1.6–5.7) | 4.5 (1.4–6.7) | 0.603 |
|  |  | 48 h | 2.4 (1.3–5.4) | 4.7 (1.3–7.2) | 0.285 |
|  |  | 72 h | 2.5 (1.3–5.6) | 4.8 (1.3–6.8) | 0.297 |
|  | IL-8, pg/mL | |  |  |  |
|  |  | Baseline | 13.9 (3.4–61.3) | 43.2 (6.3–70.2) | 0.299 |
|  |  | 24 h | 22.8 (2.6–57.1) | 46.2 (6.1–63.5) | 0.488 |
|  |  | 48 h | 14.8 (1.9–65.6) | 41.3 (7.0–67.5) | 0.488 |
|  |  | 72 h | 14.6 (2.6–56.7) | 43.3 (7.2–63.1) | 0.184 |
|  | IL-10, pg/mL | |  |  |  |
|  |  | Baseline | 53.1 (19.7–241.4) | 164.7 (22.4–315.0) | 0.326 |
|  |  | 24 h | 92.4 (20.0–256.1) | 172.7 (36.1–283.8) | 0.470 |
|  |  | 48 h | 82.8 (20.0–246.5) | 164.9 (24.3–286.8) | 0.371 |
|  |  | 72 h | 60.9 (18.5–244.9) | 186.7 (20.8–278.2) | 0.686 |
|  | MIP-2, pg/mL | |  |  |  |
|  |  | Baseline | 4.8 (3.7–8.3) | 6.6 (3.8–9.9) | 0.402 |
|  |  | 24 h | 5.2 (3.5–8.0) | 6.8 (3.7–9.0) | 0.386 |
|  |  | 48 h | 6.4 (3.6–8.1) | 6.4 (3.7–9.5) | 0.436 |
|  |  | 72 h | 4.5 (3.5–7.6) | 6.1 (3.6–9.0) | 0.470 |
| Adverse events | | | | |  |
|  | Severe intolerance of NPPV, n (%) | | 0 (0.0) | 1 (8.3) | >0.999 |
|  | Abdominal distention, n (%) | | 3 (25.0) | 1 (8.3) | 0.590 |
|  | Abdominal circumference, cm^✝^ | |  |  |  |
|  |  | Baseline | 94.3 ± 12.4 | 101.5 ± 16.0 | 0.228 |
|  |  | 2 h | 94.1 ± 12.7 | 101.6 ± 15.9 | 0.217 |
|  |  | 6 h | 94.3 ± 12.3 | 100.7 ± 15.9 | 0.281 |
|  |  | 24 h | 94.2 ± 12.5 | 101.2 ± 15.7 | 0.240 |
|  |  | 48 h | 94.5 ± 12.6 | 100.8 ± 15.8 | 0.290 |
|  |  | 72 h | 94.0 ± 13.2 | 101.6 ± 16.0 | 0.218 |
| Data are presented as means ± standard deviations, medians (25th–75th percentiles), or frequencies (percentages) of patients as appropriate  NPPV noninvasive positive pressure ventilation, TNF tumour necrosis factor, IL interleukin, MIP macrophage inflammatory protein  *Determined over the last 10 min of the tracings of oesophageal pressure, airway pressure, and flow within 24 h after randomisation  ^✝^No significant differences were observed within the two groups | | | | | |

| **Table S4 Clinical outcomes** | | | | |
| --- | --- | --- | --- | --- |
| Variable | | High-intensity NPPV (n = 12) | Low-intensity NPPV (n = 12) | p value |
| Need for intubation, n (%) | | 0 (0.0) | 1 (8.3) | >0.999 |
| Successful weaning of NPPV, n (%) | | 0 (0.0) | 7 (58.3) | 0.005 |
| Death, n (%) | |  |  |  |
|  | In the ICU | 0 (0.0) | 1 (8.3) | >0.999 |
|  | At day 28 | 0 (0.0) | 1 (8.3) | >0.999 |
|  | At day 90 | 0 (0.0) | 1 (8.3) | >0.999 |
| Live ICU discharge, n (%) | | 11 (91.7) | 11 (91.7) | >0.999 |
| Live ICU discharge within 28 days after randomisation, n (%) | | 10 (83.3) | 11 (91.7) | >0.999 |
| ICU length of stay, days | | 10.4 ± 8.9 | 8.9 ± 3.1 | 0.591 |
| ICU length of stay after randomisation, days | | 10.3 ± 8.8 | 8.6 ± 3.1 | 0.545 |
| ICU-free days within 28 days after randomisation, days | | 17.8 ± 8.8 | 19.4 ± 3.1 | 0.545 |
| ICU costs, ×10^3^ RMB | | 30.5 (19.4–36.8) | 29.7 (20.2–37.4) | 0.862 |
| Suggestion for home NPPV with ICU discharge, n (%) | | 11 (91.7) | 4 (33.3) | 0.009 |
| Home NPPV at day 90, n (%) | | 11 (91.7) | 6 (50.0) | 0.069 |
| 90-day hospital readmission, n (%) | | 2 (16.7) | 4 (33.3) | 0.640 |
| Data are presented as means ± standard deviations, medians (25th–75th percentiles), or frequencies (percentages) of patients as appropriate  NPPV noninvasive positive pressure ventilation, ICU intensive care unit | | | | |
